# Interfacial Interactions between *Escherichia coli* and Polystyrene Nanoplastics: a Physicochemical Perspective

**DOI:** 10.1021/acs.jpcb.5c08029

**Published:** 2026-02-11

**Authors:** Monika Naumowicz, Joanna Kotyńska, Marcin Zając, Piotr Deptuła, Joanna Breczko, Robert Bucki, Izabela Święcicka

**Affiliations:** † Laboratory of Bioelectrochemistry, Department of Physical Chemistry, Faculty of Chemistry, 49663 University of Bialystok, 1K K. Ciolkowski Str., 15-245 Bialystok, Poland; ‡ Doctoral School of Exact and Natural Sciences, University of Bialystok, 1K K. Ciolkowski Str., 15-245 Bialystok, Poland; § Department of Medical Microbiology and Nanobiomedical Engineering, Medical University of Bialystok, 2C A. Mickiewicz Str., 15-222 Bialystok, Poland; ∥ Laboratory of Materials Chemistry, Department of Physical Chemistry, Faculty of Chemistry, University of Bialystok, 1K K. Ciolkowski Str., 15-245 Bialystok, 15-245 Bialystok, Poland; ⊥ Department of Microbiology and Biotechnology, Faculty of Biology, University of Bialystok, 1J K. Ciolkowski Str., 15-245 Bialystok, Poland; # Laboratory of Applied Microbiology, Department of Microbiology and Biotechnology, Faculty of Biology, University of Bialystok, 1J K. Ciolkowski Str., 15-245 Bialystok, Poland

## Abstract

Nanoplastics are increasingly recognized as emerging environmental contaminants, yet the physicochemical mechanisms governing their interactions with bacterial cells remain insufficiently understood. In this study, we investigated the interactions between Gram-negative *Escherichia coli* and polystyrene nanoparticles (PS and PS-NH_2_; 100 and 200 nm) using electrophoretic light scattering (ELS), Fourier-transform infrared (FTIR) spectroscopy, and atomic force microscopy (AFM). Zeta potential measurements revealed concentration-, pH-, and time-dependent shifts in the electrokinetic behavior of bacteria–nanoparticle mixtures, reflecting composite signals arising from nanoparticle attachment and surface-level interactions rather than direct measurements of bacterial surface charge. FTIR and AFM analyses confirmed nanoparticle surface adhesion and localized envelope perturbations; however, evidence for nanoparticle penetration remained indirect and subject to methodological limitations. Microbiological assays showed growth inhibition at nanoparticle concentrations ≥50 μg/mL, but no bactericidal activity was conclusively confirmed under the applied conditions. Overall, the results demonstrate that polystyrene nanoparticles induce measurable physicochemical and sublethal biological effects on *E. coli* without reaching cytotoxic thresholds, underscoring the importance of cautious interpretation when linking nanoparticle-induced surface perturbations to biological outcomes.

## Graphical abstract

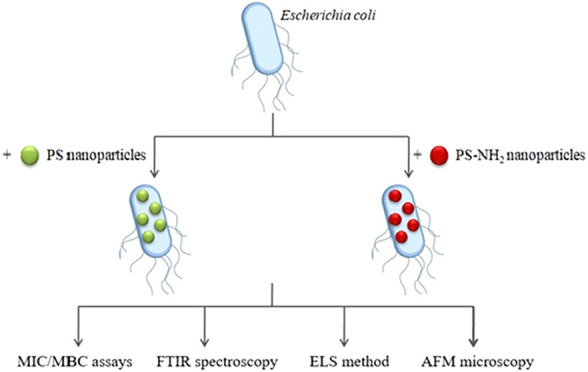

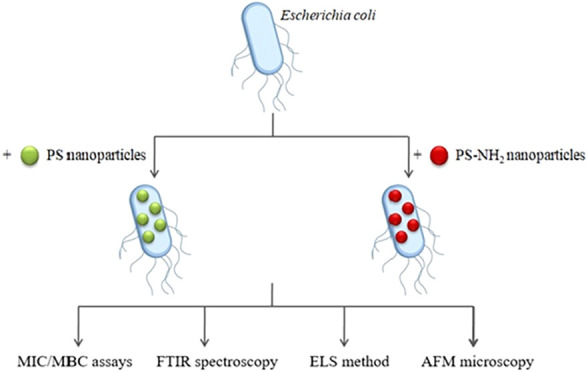

## 1 Introduction

Plastic materials are synthetic polymers engineered with additives to enhance mechanical durability, radiation resistance, and protection against microbial degradation. Once released into the environment, plastic debris undergoes physicochemical aging through photodegradation, biodegradation, hydrolysis, and mechanical abrasion, processes that progressively alter its surface chemistry.^1^ Weathering often generates negatively charged surfaces via oxidation and formation of carboxyl groups, whereas positively charged surfaces may arise from amine-containing modifications or polymer hydrolysis.^2^

Plastic waste fragmentation generates particles spanning a broad size range, including nanoplastics (NPs). Although no universally accepted definition exists, many authors consider particles below 100 ^1,3^ or 1000 nm^4,5^ as nanoplastics, with physicochemical criteria increasingly emphasized over purely dimensional ones. The lack of standardized definitions and analytical protocols complicates both their reliable characterization and the assessment of their environmental toxicity.^6^ Direct quantification of nanoplastics in environmental matrices remains technically challenging, and reported concentration ranges vary substantially between studies. As a result, laboratory investigations often employ concentration ranges exceeding current field estimates in order to elucidate interaction mechanisms that may also occur at lower, environmentally relevant levels but remain difficult to resolve experimentally.

Polystyrene (PS) is among the most widely distributed polymers, detected in soils,^7^ sediments,^8^ freshwater,^9,10^ marine systems,^11,12^ and remote polar regions.^13,14^ Its prevalence reflects extensive industrial use in lightweight plastics and foams.^2,15^ Despite ubiquity, the impacts of PS particles on microorganisms, mainly bacteria at the base of many food webs, remain insufficiently resolved.^16,17^

From a physical chemistry perspective, nanoplastic–bacteria interactions are governed by interfacial forces acting at the nano- and microscale. Electrostatic interactions, van der Waals attraction, and steric effects collectively determine particle attachment, aggregation behavior, and surface association.^18^ Classical colloid and interface theories, including DLVO-type concepts, provide a helpful framework for interpreting such interactions, although biological surfaces introduce additional complexity.

Bacterial cells cannot be treated as rigid colloidal particles. Instead, they represent soft, heterogeneous entities composed of charged polymers, proteins, and polysaccharides extending into the surrounding medium. As a result, electrokinetic measurements performed on bacteria–nanoparticle systems yield composite signals reflecting contributions from both components rather than solely the intrinsic bacterial surface charge. This complexity necessitates cautious interpretation of apparent zeta potential values.

The surface charge of bacterial cells varies significantly depending on the pH of the surrounding environment. It arises from the presence of negatively charged groups (eg carboxyl, phosphoryl, or sulfhydryl groups from carbohydrates and proteins) or positively charged groups (amino groups of amino acids, amino sugars, or lipids).^18^ These acid–base functional groups are linked to peptidoglycans, teichoic acids, and teichuronic acids present in the wall of Gram-positive bacteria and to lipopolysaccharides, phospholipids, and proteins in the outer membrane of Gram-negative bacteria, such as *Escherichia coli*.^19^ Under physiological conditions, most bacteria exhibit a net negative surface charge.^20^

Determining cell-surface charge typically requires time-intensive experiments that explore various methodological approaches. However, this physicochemical parameter can be evaluated using the zeta (ζ) potential, representing the electrical potential at the interface between the bacterial surface and the surrounding aqueous environment.^21^ In many contexts, the zeta potential is a critical parameter that, on the one hand, contributes to maintaining cell function and, on the other hand, provides valuable information on cell surface properties.^22–24^ The ζ potential can be estimated by measuring the electrophoretic mobility of cells in an electric field without destruction of cells or alteration of the surface composition.^25^ An electrophoretic light scattering (ELS) technique has demonstrated considerable utility in various physiological studies, including the characterization of the surface properties of bacteria.^20,26^ ELS is favored by researchers for its speed and simplicity in estimating zeta potentials.

Most studies exploring plastic toxicity rely on commercially available spherical PS nanoparticles.^27^ While these model systems allow controlled physicochemical evaluation, their environmental relevance is limited by the complexity and heterogeneity of real-world plastic fragments. In fact, commercial PS particles constitute only a small subset of plastics present in the environment.^28^ However, given the limited data on plastic environmental toxicity, existing studies on the biological effects of spherical PS offer valuable mechanistic insight into the primary factors driving plastic toxicity across different organism groups, while simultaneously helping to identify key knowledge gaps and guide future research priorities.

Previous studies have reported growth inhibition, oxidative stress, membrane damage, and metabolic disturbances in bacteria exposed to polystyrene nanoplastics.^17,29,30^ However, it is important to distinguish between growth inhibition, sublethal stress responses, and confirmed cytotoxic or bactericidal effects, as these outcomes reflect fundamentally different biological and physicochemical mechanisms.

Despite the rapidly growing body of literature on nanoplastic–microorganism interactions, significant gaps remain in the physicochemical understanding of these systems. Many previous studies have focused primarily on toxicological end points or qualitative observations, often without explicitly linking nanoparticle surface properties, concentration-dependent behavior, and electrokinetic responses to nanoscale interaction mechanisms. Consequently, it remains unclear to what extent the reported bacterial responses arise from interfacial association processes governed by colloidal forces rather than from cytotoxic or penetrative effects. Moreover, experimental conditions such as ionic strength and electrolyte composition, which strongly influence colloidal interactions, are often insufficiently discussed. Low ionic strength conditions are frequently employed to enhance electrokinetic sensitivity, yet their implications for environmental relevance must be explicitly acknowledged.

In this study, we address these gaps by systematically analyzing of the interactions between *E. coli* cells and polystyrene nanoparticles with different surface functionalities under controlled aqueous conditions. By integrating electrokinetic measurements, spectroscopic analysis, and atomic force microscopy, we aim to elucidate adsorption-driven, surface-level interaction mechanisms while explicitly accounting for methodological limitations. This integrated approach allows for a cautious yet mechanistically grounded interpretation of bacteria–nanoplastic interactions without overestimating biological impact.

## 2 Experimental Section

### 2.1 Polystyrene Nanoplastics and Reference Materials

Two particle sizes (100 and 200 nm) of commercially available polystyrene, both nonfunctionalized (PS) and amino-functionalized (PS-NH_2_), were used in the study. The PS nanoparticles were sourced from Sigma-Aldrich (Saint Louis, USA) under catalog numbers 43302 and 69057 for the 100 nm PS and the 200 nm PS, respectively. The 100 nm PS-NH_2_, catalog number 16586-5, was acquired from Polysciences (Hirschberg an der Bergstrasse, Germany), while the 200 nm PS-NH_2_, catalog number PA02001, was obtained from Bang Laboratories (Fishers, Indiana, USA). According to the product datasheets, the PS nanoparticle sizes were 100 and 200 nm (with a standard deviation of 0.01 μm), with particle charge densities of 1.05 g/cm^3^, and both packaged as 10% solid aqueous suspension containing 0.1–0.5% surfactant, sodium bicarbonate, and potassium sulfate. According to the manufacturers, the average diameters of the PS-NH_2_ nanoparticles were 0.112 and 0.200 μm (within a 0.190–0.210 μm range). The particle charge density, ranging from approximately 1.05 to 1.1 g/cm^3^, was provided only for the 200 nm PS-NH_2_. The 100 nm PS-NH_2_ particles were packaged as 2.63% solid (w/v) aqueous suspensions, while the 200 nm PS-NH_2_ particles were supplied as 10.09% solids. 100 nm PS-NH_2_ suspensions contained undisclosed anionic surfactants, while 200 nm PS-NH_2_ suspensions contained ≤0.5% Tween 20 or sodium dodecyl sulfate (SDS), along with 0.09% sodium azide. Prior to biological and physicochemical experiments, nanoparticle suspensions were subjected to repeated washing and centrifugation steps, then resuspended in surfactant-free electrolyte solutions to minimize the presence of residual stabilizing agents.

Sodium chloride, potassium bromide, sodium hydroxide, glycerol, hydrochloric acid, phosphate buffered saline (PBS), and poly-l-lysine were sourced from Sigma-Aldrich (Saint Louis, USA). Ethanol was obtained from Merck (Darmstadt, Germany). Microbiological media, including Mueller-Hinton broth (MHB), Mueller-Hinton agar (MHA), Luria–Bertani broth (LB), nutrient agar, and brain heart infusion (BHI) agar, were purchased from Oxoid Ltd. (Basingstoke, U.K.).

Electrolyte solutions and cleaning procedures were performed using ultrapure water (resistivity of 18.2 MΩ) from the HLP 5UV System (Hydrolab, Hach Company, Loveland, USA).

### 2.2 Characterization of Polystyrene Nanoparticles

DLS and ELS were employed to characterize both nonfunctionalized and amino-functionalized polystyrene NPs using a Zetasizer Nano-ZS (Malvern Instruments Ltd., Malvern, United Kingdom). The particle sizes, the polydispersity index (PDI) of the suspension, and the particle zeta potential (ζ) were determined using a 1 mL DTS1061 folded capillary cell (Malvern Instruments). Initially, the nanoparticles were diluted in 0.3 mM NaCl to achieve final concentrations of 0.4, 2, 20, or 100 μg/mL. The suspensions were then homogenized by sonication immediately before testing with a probe sonicator for 20 min to minimize aggregation (Techpan, Warsaw, Poland). All measurements were carried out at pH 7.4 and 25 °C.

### 2.3 Bacterial Preparation Technique

The ATCC 11229 strain of *E. coli* was acquired from the American Type Culture Collection (ATCC, Manassas, USA). Bacterial cells preserved at −80 °C in LB broth and glycerol storage medium (1:1 ratio) were streaked onto nutrient agar, incubated overnight at 37 °C, and stored at 4 °C for future use. A single colony was harvested from the agar plate, transferred to Mueller-Hinton broth (MHB) and cultured at 37 °C for 24 h. The bacterial suspension was prepared by resuspending the pellet in a background solution (either distilled, autoclaved water or a NaCl solution with the same ionic strength as in the respective experiment). The bacterial suspensions were centrifuged at 8000 rpm for 20 min; the supernatant was discarded, and the pellets were washed 5 times with the background solution to ensure thorough removal of residual growth medium. The optical density of the final dispersion (OD_600_) was measured using a V-670 spectrophotometer (Jasco Corp., Tokyo, Japan) and ranged from 0.10 to 0.12.

### 2.4 Determination of the Minimum Inhibitory Concentration (MIC) and the Minimum Bactericidal Concentration (MBC)

MIC values were established according to the CLSI protocol.^31^ Briefly, PS or PS-NH_2_ nanoparticle solutions were prepared in Mueller-Hinton broth (MHB) and serially diluted in a 96-well microtiter plate of U bottom to final concentrations of 1:2, 1:4, 1:16, 1:64, and 1:256 in a total volume of 100 μL of *E. coli* cultures grown overnight in MHB at 37 °C with agitation at 160 rpm, and subsequently adjusted to a final optical density of 0.2–0.3 at 600 nm, measured using a V-670 spectrophotometer (Jasco Corp.). A 100 μL aliquot of the bacterial suspension was added to each well containing diluted NPs. Microtiter plates were incubated overnight at 37 °C. The minimum inhibitory concentration (MIC) was defined as the lowest nanoparticle concentration at which no visible increase in turbidity relative to the control was observed. Importantly, MIC values obtained under these conditions indicate growth inhibition and do not constitute direct evidence of bactericidal or cytotoxic effects.

All assays were carried out in quadruples, and the results were averaged.

To determine the MBC values of the polystyrene nanoparticle extracts, 5 μL of the overnight culture from each well containing NP at concentrations equal to or exceeding the MIC was aseptically transferred to BHI agar plates using a sterile plastic spreader and incubated at 37 °C overnight. MBC was defined as the lowest extract concentration at which no bacterial colonies were visually detected in the agar plates.

It should be noted that in nanoparticle-containing suspensions, increased turbidity and light scattering may arise from the physical presence of nanoparticles rather than from bacterial growth. In addition, nanoparticle aggregation and adsorption to bacterial cells may influence apparent colony recovery. Therefore, MBC values labeled as “not determined (n.d.)” do not necessarily indicate the absence of bactericidal activity but rather reflect methodological limitations in distinguishing between growth inhibition and cell killing under these experimental conditions.

Each experiment was carried out in quadruplicate, and the average results were reported. In the control setup, bacteria grown in BHI without NP were the positive control.

### 2.5 Measurements of Zeta Potential

Electrophoretic mobility measurements were performed to evaluate the electrokinetic behavior of bacterial cells in the presence and absence of polystyrene nanoparticles. Measurements were conducted (using a Zetasizer Nano ZS analyzer (Malvern Instruments Ltd.)) in aqueous NaCl solutions at controlled ionic strength and pH.

For this, 0.2 mL of freshly prepared bacterial suspension was mixed with 0.8 mL of the desired NaCl concentration. The pH was adjusted to the target value (3–11) by adding HCl or NaOH solutions dropwise, with the same ionic strength as the cell suspension. The mixture was stirred and conditioned until the pH stabilized. The resulting suspension was then applied to a clear disposable zeta cell with gold-plated electrodes (DTS 1070, Malvern Instruments Ltd.), and the measurement was taken. Between measurements, the electrodes were thoroughly rinsed with ethanol and deionized water, then with the bacterial suspension. The zeta potential was determined at the specified pH and a temperature of 25 °C by averaging six consecutive Zetasizer readings (each consisting of 100–200 runs). The results for each condition represent the mean of three independent measurements conducted on separately prepared samples to ensure reproducibility.

Additionally, we investigated the effect of ionic strength on the zeta potential of *E. coli* cells. To do this, the *E. coli* suspension was washed three times with an appropriate concentration of NaCl and resuspended in sodium chloride solutions at concentrations of 0.3, 30, 100, or 155 mM. The impact of treating bacteria with PS and PS-NH_2_ nanoparticles was evaluated by adding NP to 0.3 mM NaCl, sonicating for 20 min and mixing with the bacterial suspension to achieve final NP concentrations of 0.4, 2, 20, and 100 μg/mL. The mixture was then incubated at 25 °C in a shaking water bath for 20 min before ζ determination. To study the effect of exposure time to polystyrene nanoparticles on the zeta potential of cells, suspensions of bacteria and NPs were prepared at selected concentrations in 0.3 mM NaCl and measured immediately after preparation and after 1 and 3 h of incubation in the shaking bath.

In mixed bacteria–nanoparticle suspensions, the measured zeta potential reflects an apparent composite electrokinetic signal arising from the combined contributions of bacterial cells, free nanoparticles, and surface-associated nanoparticles.

Bacterial cells were therefore treated conceptually as soft, heterogeneous particles rather than rigid colloids. In such systems, adsorbed nanoparticles and polymeric surface components contribute to the measured electrokinetic response, and the resulting zeta potential should be interpreted as a relative indicator of changes in interfacial electrokinetic behavior rather than a direct measure of the intrinsic bacterial surface charge.

Low ionic strength conditions were intentionally selected to enhance electrokinetic sensitivity by minimizing compression of the electrical double layer. While this approach improves the detection of interaction-induced changes, it does not fully represent environmentally relevant salinities, a limitation that is explicitly accounted for in data interpretation.

### 2.6 Fourier Transform Infrared Spectroscopy Analysis

Fourier-transform infrared (FTIR) spectroscopy was used to probe changes in bacterial surface-associated functional groups following nanoparticle exposure.

FTIR measurements were performed in transmission mode using a Nicolet 6700 spectrometer (Thermo Scientific, Madison, WI, USA). To prepare samples, the *E. coli* cell suspensions were first centrifuged at 14,000 rpm for 20 min and washed 5 times with distilled water. An appropriate volume of the NP stock solution was then added to the bacterial suspension to achieve a concentration of 100 μg/mL. After a 2-h exposure of *E. coli* to polystyrene nanoparticles under stirring conditions, the samples were frozen in liquid nitrogen and lyophilized for 24 h in a freeze-dryer (Christ α 1–2 LD plus double chamber, Osterode am Harz, Germany) at 0.013 mbar. The resulting powders were mixed with potassium bromide (KBr), ground in an agate mortar, and compressed into pellets, which were then placed in the spectrophotometer’s measurement chamber for analysis.

It is emphasized that FTIR provides ensemble-averaged spectral information and does not resolve spatial localization of nanoparticles or confirm intracellular internalization. Observed spectral changes were therefore interpreted as indirect indicators of surface-level interactions rather than evidence of penetration.

### 2.7 Measurements of the Atomic Force Microscope

Atomic force microscopy (AFM) was employed to characterize nanoscale surface features and interaction forces of bacterial cells following nanoparticle exposure. Imaging and force spectroscopy were performed under liquid conditions to preserve native cell surface properties. On the day before the measurements, 100 μL of a 0.01% poly l-lysine solution was incubated with cut mica slices. A single colony of *E. coli* that grew overnight on nutrient agar was resuspended in PBS (OD600: ∼ 0.1), followed by incubation with 2, 20, and 100 μg/mL of PS and PS-NH_2_ nanoparticles (100 and 200 nm in diameter) at 37 °C for 0.5 h. Then, 100 μL of bacterial suspension was transferred to the mica surface previously functionalized with poly-l-lysine and incubated for 30 min at 37 °C in PBS.

Images of bacterial cells were collected using a NanoWizard 4 BioScience atomic force microscope (AFM; JPK/Bruker, MA, USA) equipped with a liquid cell. Due to the lateral forces during contact mode scanning, AFM was operated in QI mode (JPK QITM mode - Quantitative Imaging). Silicon nitride AFM cantilevers (Bruker, MSCT-E) with a nominal spring constant of 0.1 N/m and a measured spring constant in the range of 0.13–0.18 N/m were used using the thermal tuning method. Bacterial cells were visualized using an optical microscope, and then topographic maps of 5 μm × 5 μm were generated with a resolution of 128 pixels per line under wet conditions. QI maps were taken in several AFM modes. We took (i) height mode to show the topography of bacterial cells (using an edge detector system), (ii) slope mode to show stiffness maps of mica and bacterial surfaces, and (iii) adhesion mode to show the adhesion forces between the AFM cantilever and bacterial cell surfaces. However, in regions with dense nanoparticle coverage, accurate height determination was limited by tip convolution effects and surface heterogeneity. Consequently, AFM-derived parameters were interpreted qualitatively and comparatively rather than as absolute structural descriptors.

### 2.8 Statistical Analysis

Data are displayed as average ± standard deviation (SD). Statistical significance was assessed using one-way analysis of variance (ANOVA) followed by Scheffe’s *F* test; a *p*-value of less than 0.05 was considered statistically significant. The following significance levels were used: * *p* < 0.05; ** *p* < 0.01; *** *p* < 0.001; **** *p* < 0.0001.

## 3 Results and Discussion

### 3.1 Role of pH and Electrolyte Concentration in the Bacterial Zeta Potential

An analysis of the ATCC 11229 *E. coli* strain’s tolerance to pH variations in its growth environment was conducted before the primary electrokinetic measurements. The assays involved incubating the bacterium on LB agar for 24 h at pH values between 3 and 11. The bacterium exhibited robust growth in media with pH values between 4 and 10, whereas growth at pH 3 and 11 was significantly reduced. Consequently, the pH range for zeta potential measurements was extended by only one unit beyond the optimal growth range (pH 3–11) to ensure physiological relevance while avoiding excessive stress to the cells.

Figure 1 illustrates the ζ values of *E. coli* cells measured in sodium chloride solutions at different concentrations (0.3, 30, 100, and 155 mM) using ELS, obtained as a function of pH. The surfaces of the bacterial cells displayed a negative charge within the studied pH range (except at pH 3.0 for the lowest NaCl concentration), a property common to most microorganisms.^32^ Additionally, the ζ values were influenced by changes in pH and sodium chloride concentration. The zeta potential became less negative as the electrolyte concentration increased, which can be attributed to a reduction in the electrical double layer thickness and a decrease in the effective surface charge at higher ionic strength.^33^ Furthermore, ζ values gradually increased with decreasing pH, with a significant reduction occurring at acidic pH levels. This effect was due to neutralization of the negatively charged cell surface by excess protons in the surrounding environment. Under natural conditions, *E. coli* can survive for several hours without growing at pH 2.0, whereas its acid growth threshold is pH 4.0 in nutrient-rich media and pH 4.5 in minimal media.^34^ Acidic pH in natural environments alters the dissociation density of the surface groups of the bacterium.

**Figure 1:**
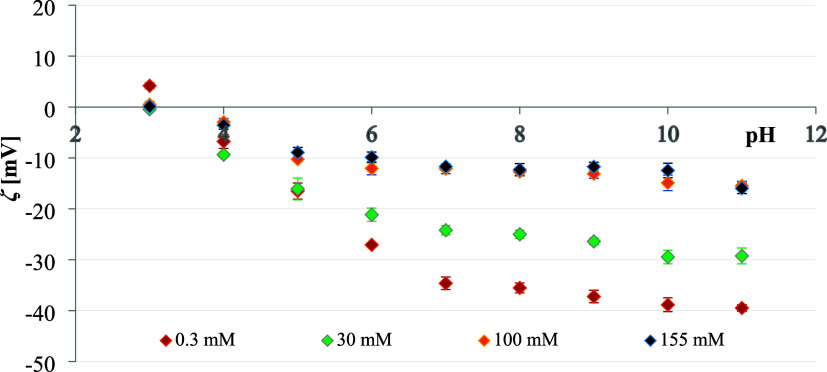
Zeta potential of ATCC 11229 *E. coli* strain cells was plotted against pH at different NaCl concentrations.

The isoelectric point provides valuable information about the surface charge of microorganisms, indicating the pH at which the net surface charge equals zero.^35^ As shown in Figure 1, the isoelectric point of *E. coli* was observed at pH ≈ 3, which aligns with values documented by Hayashi et al.^36^ Notably, the position of the isoelectric point remained unchanged across the examined electrolyte concentrations, indicating that NaCl functioned as an inert electrolyte, without specific adsorption to the bacterial surface. This behavior is consistent with observations in other bacterial systems.^34,35,37^

The zeta potential is widely regarded as a key parameter for evaluating the stability of colloidal dispersions.^38^ Generally, a ζ value of ±25 mV distinguishes low-charged from high-charged surfaces.^39^ The data shown in Figure 1, therefore, enable identification of the most suitable medium for electrokinetic analysis of *E. coli*. A 0.3 mM NaCl solution was selected as the optimal medium because it yielded the highest absolute zeta potential values, thereby ensuring maximum suspension stability. Accordingly, all electrokinetic interaction experiments were conducted at an ionic strength of 0.3 mM NaCl. This low electrolyte concentration was selected to ensure colloidal stability of both bacterial cells and polystyrene nanoparticles and to maximize the sensitivity of electrophoretic mobility measurements by minimizing compression of the electrical double layer. At higher ionic strengths, increased electrostatic screening reduces the magnitude of electrokinetic responses and may obscure interaction-induced shifts in zeta potential. Therefore, the chosen conditions allow for more precise detection of relative changes in electrokinetic behavior upon nanoparticle exposure, while acknowledging that they do not fully represent environmentally relevant salinities.

To assess the robustness of the observed trends, additional measurements were performed at higher NaCl concentrations (30, 100, and 155 mM; Figure 1). While the absolute magnitude of zeta potential shifts decreased with increasing ionic strength, the qualitative trends remained consistent, indicating that the underlying interaction mechanisms persist under more saline conditions.

It should be emphasized that zeta potential values reported in this section represent apparent electrokinetic properties of the system under defined physicochemical conditions. They serve as relative indicators for comparing interaction trends rather than as absolute descriptors of intrinsic bacterial surface charge.

From a physicochemical perspective, the observed dependence on ionic strength is consistent with classical colloidal interaction theory, in which electrostatic repulsion mediated by the electrical double layer governs nanoparticle–cell interactions. These considerations provide a mechanistic framework for interpreting the concentration- and time-dependent electrokinetic trends discussed in subsequent sections.

### 3.2 Characterization of Polystyrene Nanoparticle Size, Polydispersity, and Zeta Potential

The physicochemical properties of polystyrene nanoparticles and *E. coli* cells were characterized under the same aqueous conditions as those used in the interaction experiments. Nanoparticle hydrodynamic size and electrophoretic mobility were determined to verify colloidal stability and surface charge characteristics prior to exposure to bacterial cells.

As a first step, the PS-100, PS-200, PS-NH_2_-100, and PS-NH_2_-200 nanoparticles used in the study were thoroughly characterized for their size and polydispersity index (PDI) using dynamic light scattering (DLS). Measurements were performed in 0.3 mM sodium chloride solution at pH 7.4 using a Zetasizer Nano ZS. The results for NPs at a concentration of 100 μg/mL are presented graphically in Figure 2 and numerically in Table 1. Data for the other concentrations examined in the study (0.4, 2, and 20 μg/mL) are provided in Table S1.

**Figure 2:**
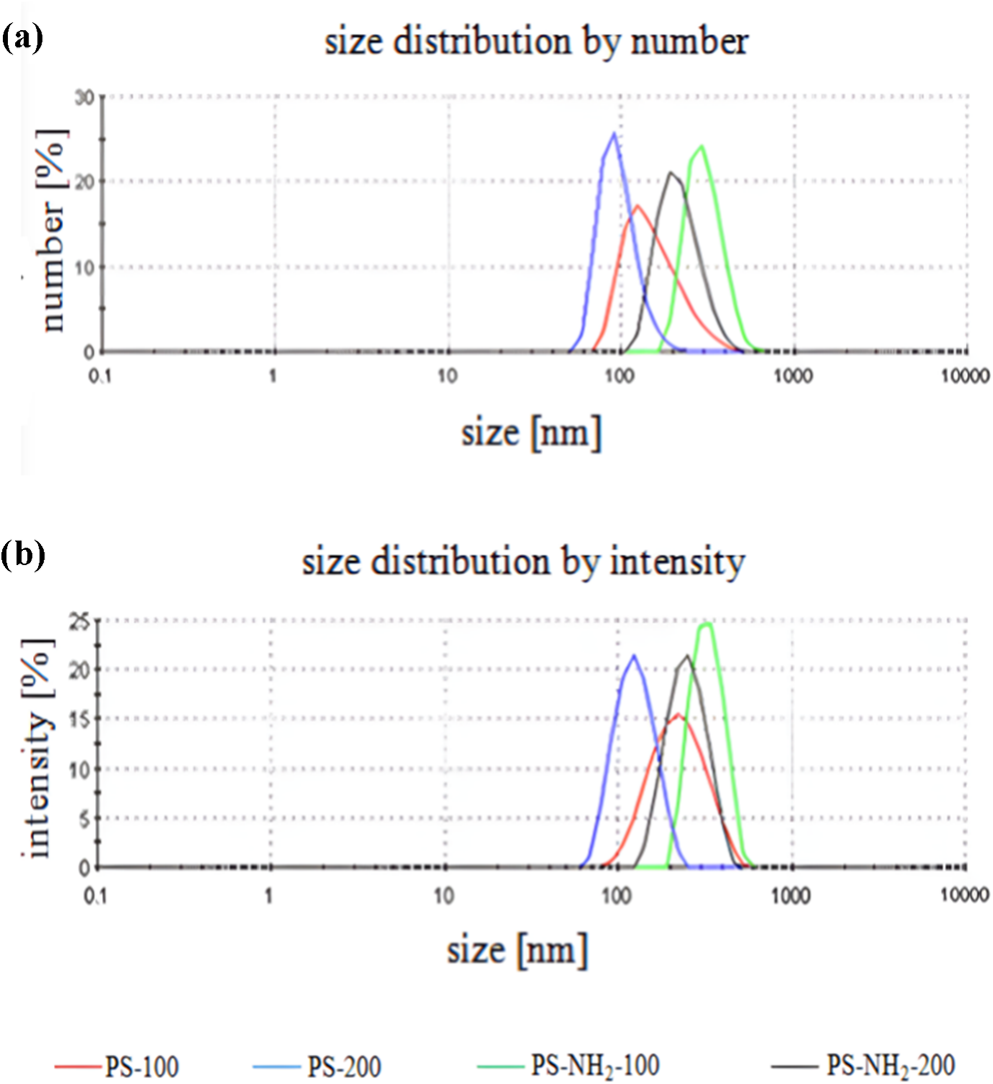
Polystyrene nanoparticle size distribution according to (a) number and (b) intensity in 0.3 mM NaCl (*C*_NPs_ = 100 μg/mL, pH = 7.4).

**Table 1:** Characterization of Polystyrene (PS) Nanoparticles Tested (*C*_NPs_ = 100 μg/mL, 0.3 mM NaCl, pH = 7.4)

Nanoparticle	Size by number [nm]	Size by intensity [nm]	PDI^a^	Zeta potential [mV]
PS-100	158.10 ± 62.80	231.60 ± 80.84	0.105	–14.70 ± 0.29
PS-200	303.90 ± 71.15	329.40 ± 68.77	0.077	–10.33 ± 0.32
PS-NH_2_-100	97.20 ± 24.68	127.20 ± 32.07	0.051	–27.98 ± 1.07
PS-NH_2_-200	219.10 ± 60.63	253.70 ± 63.15	0.008	–21.55 ± 1.01

a PDI, polydispersity index. PS-NH_2_ refers to the amino-functionalized PS.

The size distribution plots of polystyrene NPs by number and intensity (Figure 2) revealed the formation of monomodal fractions. As summarized in Table 1, the particle size distributions by number for the smaller nanoparticles meet the manufacturer’s specifications, confirming their relative stability. The diameters measured by number are 158.10 ± 62.80 nm for PS and 97.20 ± 24.68 nm for PS-NH_2_, consistent with the sizes of the particles suspended in 155 mM NaCl.^38^ By intensity, their sizes are 231.60 ± 80.84 nm and 127.20 ± 32.07 nm, respectively. For 200 nm PS, the diameters are recorded as 303.90 ± 71.15 nm (by number) and 329.40 ± 68.77 nm (by intensity). Similarly, 200 nm PS-NH_2_ particles are measured as 219.10 ± 60.63 nm (by number) and 253.70 ± 63.15 nm (by intensity). These findings suggest that the diameters of 200 nm polystyrene NPs are larger than those reported by the manufacturer, likely due to a polymer layer and a hydration shell adsorbed on the nanoparticle surfaces.^41^ DLS measurements also revealed that all tested nanoparticles exhibited low polydispersity indices, indicating monodisperse, uniform distributions in 0.3 mM NaCl. On the contrary, a higher PDI could signify homogeneous aggregate formation in suspension.^42^

The zeta potential values (as an indicator of surface charge) obtained with ELS for particles at a concentration of 100 μg/mL are summarized in Table 1. In contrast, those for the other concentrations are presented in Table S1. As shown in Table 1, 100 and 200 nm PS particles exhibit slightly negative zeta potentials of −14.70 ± 0.29 mV and −10.33 ± 0.32 mV, respectively. This could be attributed to the adsorption of hydroxyl ions at the particle–water interface.^43^ Interestingly, PS-NH_2_-100 and PS-NH_2_-200 also display negative zeta potentials, measured at −27.98 ± 1.07 mV and −21.55 ± 1.01 mV, respectively. The negative ζ potential of the functionalized particles can be attributed to an insufficient density of -NH_2_ groups on their surface, which is inadequate to achieve positive electrokinetic potential values.^44^ Furthermore, polystyrene particle suspensions may contain anionic surfactants or stabilizing agents that were not considered as separate variables in the present study. According to the manufacturer, PS-NH_2_-100 contains unspecified anionic surfactants, while PS-NH_2_-200 includes SDS, which is known to contribute to the negative ζ potential. The influence of anionic surfactants on the electrokinetic properties of polystyrene particles has been reported previously^45^ and may partially affect the measured apparent ζ potential. While trace amounts of proprietary stabilizers cannot be fully excluded, their potential contribution is expected to be minor compared to the dominant colloidal interactions governing nanoparticle–bacteria association under the applied experimental conditions.

It should be noted that electrophoretic mobility measurements of bacterial cells and nanoparticles were conducted separately to establish baseline properties. These values serve as reference points and should not be directly compared to zeta potential measurements obtained from mixed bacteria–nanoparticle suspensions, where composite electrokinetic signals are expected.

From a physicochemical perspective, both bacterial cells and nanoparticles can be considered as soft, heterogeneous colloidal entities. In particular, bacterial envelopes consist of charged polymers, proteins, and polysaccharides that extend into the surrounding medium, which affects electrokinetic behavior and distinguishes them from rigid colloidal particles. This complexity necessitates cautious interpretation of apparent zeta potential values.

The particle size and zeta potential were also evaluated during pH titration, as shown in Figures S1 and S2. The values remained broadly consistent throughout the experiments, indicating minimal aggregation under the applied conditions. This stability is essential for a meaningful interpretation of the concentration- and time-dependent interaction trends discussed in subsequent sections.

Establishing these baseline physicochemical characteristics provides the necessary framework for interpreting the adsorption-dominated interaction mechanisms, electrokinetic shifts, and adhesion phenomena analyzed in Sections 3.4–3.7.

### 3.3 Minimum Inhibitory and Minimum Bactericidal Concentrations for *E. coli* Treated with Polystyrene Nanoparticles

The MIC and MBC values for *E. coli* in the presence of 100 and 200 nm PS and PS-NH_2_ nanoparticles are presented in Table 2.

**Table 2:** Minimum Inhibitory Concentration (MIC) and Minimum Bactericidal Concentration (MBC) of Polystyrene Nanoparticles against the *E. coli* Strain ATCC 11229

Nanoparticle	MIC [μg/mL]	MBC^a^ [μg/mL]
PS-100	50	n.d.
PS-200	50	n.d.
PS-NH_2_-100	50	n.d.
PS-NH_2_-200	50	n.d.

a n.d. - no bactericidal effect.

Minimum inhibitory concentration assays consistently demonstrated growth inhibition of *E. coli* at 50 μg/mL nanoparticle concentration for both the PS and PS–NH_2_ systems. This observation indicates suppression of bacterial proliferation under the applied conditions; however, MIC values alone do not provide direct information on bactericidal or cytotoxic effects.

In contrast, MBC determination was not possible for several conditions and is therefore reported as “n.d.”. This outcome is attributed to the high turbidity and scattering of nanoparticle suspensions, which complicate the reliable discrimination between viable and nonviable bacterial populations using standard plating-based approaches. Consequently, the MBC results should be interpreted with caution and are not definitive evidence of the absence of bactericidal effects.

Such optical interference is a recognized limitation in nanoparticle–bacteria assays and can compromise the reliability of discrimination between bacteriostatic and bactericidal outcomes. Similar challenges have been reported in previous studies, where nanoparticle aggregation, adsorption to agar surfaces, and scattering effects hinder accurate colony counting.

Consequently, the absence of a clearly defined MBC value in the present study should not be interpreted as evidence of a lack of bactericidal activity. Instead, it reflects methodological constraints associated with applying conventional plate-counting techniques to nanoparticle-containing systems.

Comparable limitations in MBC determination in nanoparticle exposure studies have been discussed by Bhattacharya et al.,^46^ who demonstrated that nanoparticle-induced turbidity can obscure viability assessment, and by Qiao et al.,^27^ who highlighted the difficulty of distinguishing inhibitory from lethal effects in nanomaterial–bacteria systems without complementary viability assays.

### 3.4 Role of Polystyrene Nanoparticle Concentration in Modulating the Bacterial Zeta Potential

The zeta potential of *E. coli* cells was evaluated as a function of pH and polystyrene nanoparticle concentration using suspensions of 0.4, 2, 20, and 100 μg/mL. The upper limit was selected to cover a broad range of laboratory-relevant conditions, as higher concentrations (up to 600 μg/mL) are commonly used experimentally,^45^ whereas 250 μg/mL is considered the upper threshold for environmentally relevant levels.^46–48^ This concentration range (0.4–100 μg/mL) was chosen to systematically investigate concentration-dependent physicochemical interactions under controlled laboratory conditions rather to replicate environmental exposures directly.

Environmental concentrations of nanoplastics are highly variable and often subject to methodological uncertainty, particularly for particles below the micrometer scale. Field measurements typically range from ng/l to μg/l and often underestimate the nanoscale fraction. Accordingly, the concentrations used here should be considered experimentally relevant for mechanistic investigations of nanoparticle–bacteria interactions, rather than as direct proxies for environmental conditions. They allow identification of interaction thresholds and regimes that may guide future studies at lower, environmentally relevant concentrations. Therefore, the results are intended to advance fundamental physicochemical understanding rather than to serve as direct predictions of environmental impact.

Figure 3 shows the zeta potential values of *E. coli* cells measured at different pH levels as a function of nanoparticle concentration, indicating that exposure to PS NPs at final concentrations of 0.4 and 2 μg/mL resulted in less negative ζ values compared to the control sample (bacteria without PS NPs). This effect was significantly more pronounced for 200 nm particles than for 100 nm ones. A similar trend in the potential variation of zeta was observed for samples containing bacteria and PS-NH_2_ NP at concentrations of 0.4, 2, or 20 μg/mL. These changes are likely due to particle adhesion to the bacterial surface, resulting in a surface charge shielding effect that is more pronounced for larger particles. A comparable explanation has been suggested for interactions between negatively charged titanium dioxide nanoparticles and bacterial communities in the rhizosphere of butter crunch lettuce,^49^ as well as for neutral poly­(ethylene oxide) particles with *E. coli* and *Staphylococcus aureus*.^50^ Additionally, the attached particles may attract ions from the surrounding buffer.^51,52^

**Figure 3:**
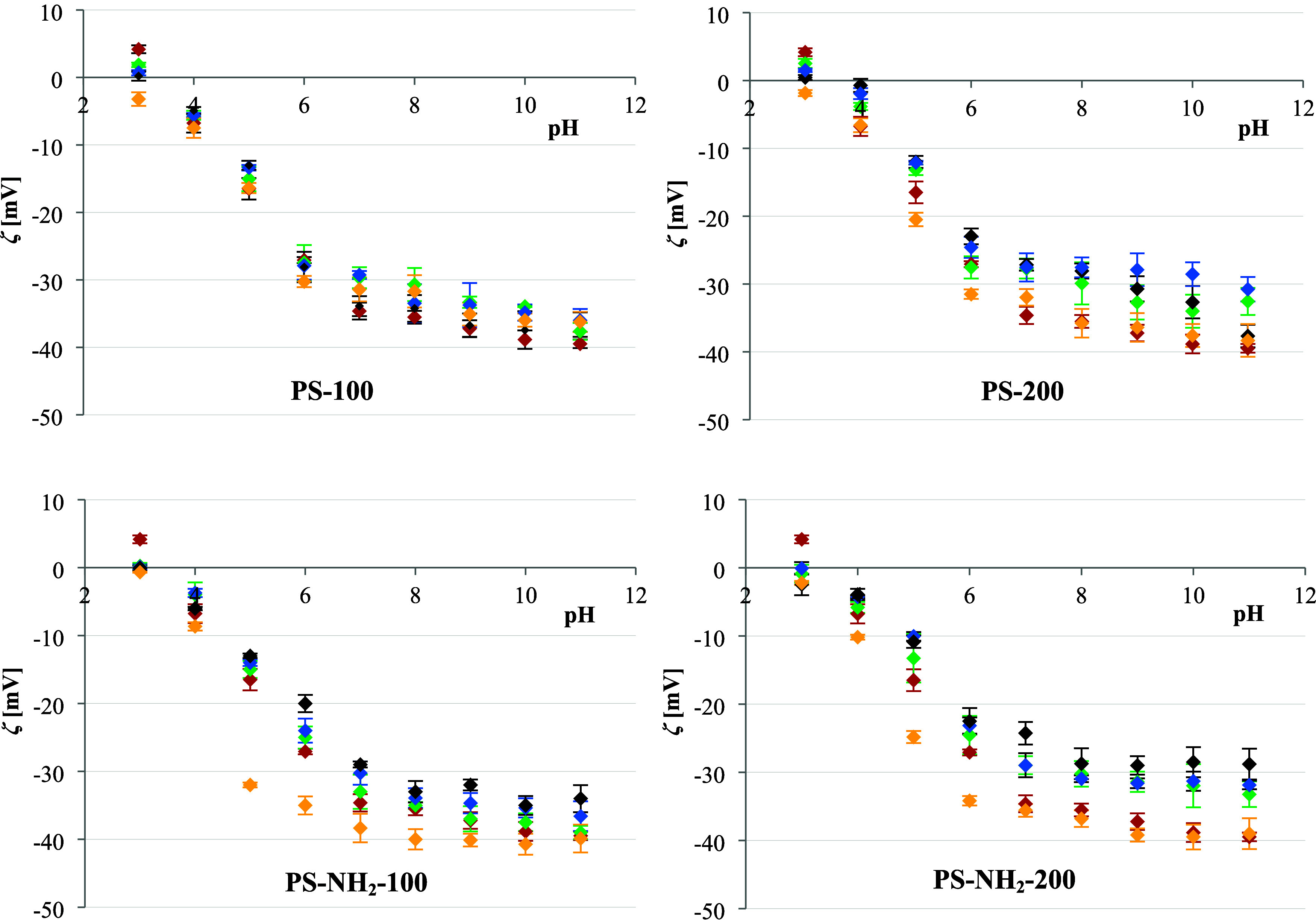
Zeta potential-pH profiles in 0.3 mM NaCl for *E. coli* strain ATCC 11229. Bacterial cells were either untreated (⧫) or exposed to polystyrene nanoparticles at concentrations of 0.4 (⧫), 2 (⧫), 20 (⧫), and 100 (⧫) μg/mL. Statistical analyzes are detailed in Tables S2 and S3.

The zeta potential measured after exposure of bacteria to PS nanoparticles at concentrations of 20 and 100 μg/mL approached values very close to those of the control, suggesting saturation of the cell surface. On the contrary, when using PS-NH_2_ particles at a concentration of 100 μg/mL, the zeta potential values were lower than those of the control sample due to the high density of positively charged groups on the nanoparticle surfaces.

Zeta potential measurements revealed concentration-dependent shifts upon exposure of *E. coli* cells to polystyrene nanoparticles. However, these values should not be interpreted as direct measurements of intrinsic bacterial surface charge. Zeta potential measurements in bacteria–nanoparticle systems are subject to inherent limitations arising from population heterogeneity, nonspherical cell shape, and the soft, deformable nature of bacterial surfaces. Consequently, the measured values represent apparent, ensemble-averaged electrokinetic responses rather than intrinsic surface charge parameters of individual cells. Because electrophoretic mobility reflects a composite electrokinetic response from both bacterial cells and associated nanoparticles, the observed shifts should be interpreted qualitatively as relative indicators of changes in interfacial electrokinetic behavior, rather than as absolute measures of bacterial surface charge or direct evidence of nanoparticle penetration. At higher pH values, the plateau-like behavior observed in Figure 3 suggests saturation of accessible surface-binding sites. This response is consistent with reversible nanoparticle adsorption rather than progressive charge neutralization and is observed in both PS and PS-NH_2_ systems. Such behavior is consistent with soft-particle electrokinetic theory, which predicts limited changes in electrophoretic mobility once the permeable surface layer becomes saturated, and has been widely reported for colloidal particles and biological cells modified by adsorbing macromolecules or nanoparticles.^53,54^ Similar saturation effects in bacterial electrokinetic responses following nanoparticle association have also been described experimentally.^55^

### 3.5 Role of Exposure Time to Polystyrene Nanoparticles in Altering Bacterial Zeta Potential

Subsequent studies focused on evaluating the zeta potential of *E. coli* as a function of pH, accounting for the cells’ exposure time to both PS and PS-NH_2_ nanoparticles. Figure 4 shows the zeta potential profiles plotted after different exposure times of *E. coli* to polystyrene particles (red points represent untreated cells, which served as the control). As shown, in the case of nonfunctionalized particles, fewer negative zeta potential values were observed with increasing exposure time. However, the zeta potential values of cells exposed to PS-NH_2_ remained similar to those observed in the control at most pH values.

**Figure 4:**
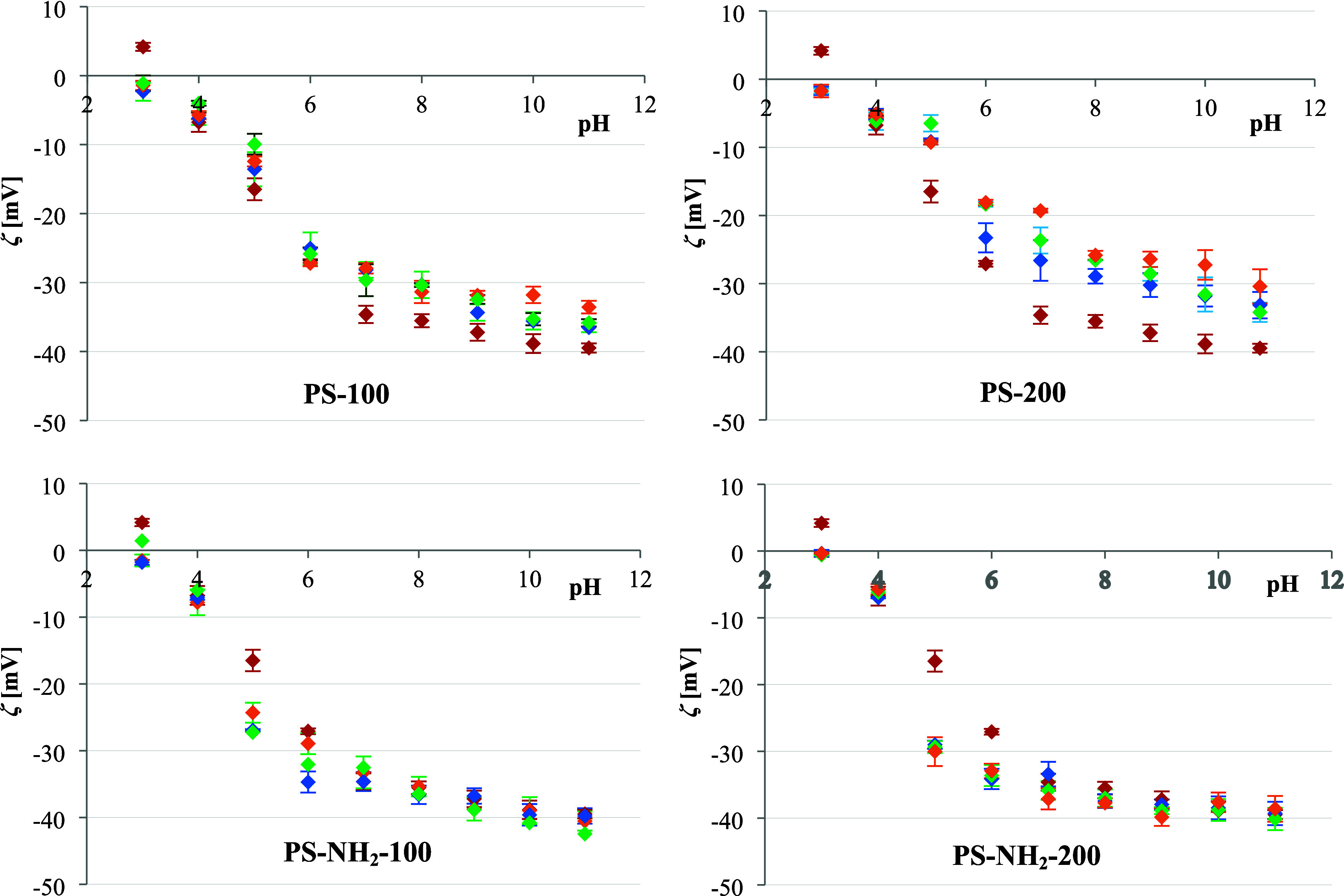
Zeta potential values of the ATCC 11229 strain of *E. coli* as a function of the pH of the electrolyte solution after various exposure times to polystyrene particles at a concentration of 100 μg/mL: (⧫) control, (⧫) 0 h, (⧫) 1 h, (⧫) 3 h. Statistical analyses are provided in Tables S4 and S5.

Zeta potential measurements revealed that the majority of electrokinetic changes occurred within the first hour of exposure to polystyrene nanoparticles, after which the zeta potential values approached a steady state and remained relatively stable over the examined time window. This rapid initial shift suggests that nanoparticle attachment to the bacterial surface is a fast process, governed primarily by physicochemical interactions rather than by slow biological adaptation or internalization mechanisms.

The subsequent stabilization of zeta potential values indicates that an equilibrium state was reached, likely corresponding to saturation of accessible surface binding sites. Such behavior is characteristic of adsorption-limited processes and has been widely reported for the attachment of colloidal particles to soft, heterogeneous surfaces, including bacterial envelopes. Notably, the absence of continuous time-dependent shifts argues against progressive nanoparticle penetration into the bacterial cell.

The surface of *E. coli* cells carries a negative charge, and electrostatic forces create a repulsive effect on the adhesion of negatively charged polystyrene nanoparticles to these surfaces.^56^ However, bacterial surfaces contain various acidic and basic functional groups that influence the electrostatic properties of the cells and ultimately regulate the likelihood of polystyrene adhesion. In this context, the surface of polystyrene nanoparticles, which contain styrene groups, can readily bond to bacteria via electrostatic and van der Waals forces.^57^ Our findings suggest that PS NP adheres to the surface of *E. coli*, while PS-NH_2_ does not show this tendency. This effect is more pronounced for particles with a diameter of 200 nm than for those with a diameter of 100 nm. The zeta potential values recorded after different exposure times of *E. coli* to polystyrene particles remained very similar, suggesting saturation of the bacterial cell surface. These observations further support the interpretation that the observed electrokinetic response reflects reversible surface-level association and partial masking of charged surface groups rather than irreversible modification of intrinsic bacterial surface charge or nanoparticle internalization. Examples of isotherms for 100 and 200 nm negatively charged nanoparticles, demonstrating Langmuir-like characteristics, can be found in the literature, providing insight into adsorption behavior through a straightforward model.^58^

### 3.6 Fourier Transform Infrared (FTIR) Spectroscopy Evaluation of Bacterial Cells and Nanoparticle Interaction

FTIR spectra of *E. coli* cells before and after exposure to PS-100, PS-200, PS-NH_2_-100, and PS-NH_2_-200 at a concentration of 100 μg/mL are shown in Figure 5. The spectrum recorded for freeze-dried bacteria (black curve) confirms the organic nature of the sample origin. The observed signals correspond to characteristic bands associated with lipids, proteins, nucleic acids, and carbohydrates on the bacterial cell membrane or in the nuclear matrix.^59,60^ The broad band at 3526 cm^–1^ was attributed to the vibrations of the −OH groups, indicating increased hydration of bacterial cells. The sequence of signals at 3030, 2943, and 2911 cm^–1^ corresponds to the stretching vibrations of the C–H, −CH_3_, and −CH_2_ groups, respectively, confirming the presence of lipids. The spectral subrange corresponding to proteins and peptides showed signals at 1653 and 1545 cm^–1^, indicating the presence of amide bands I and II.^60^ The band at 1279 cm^–1^ was assigned to the asymmetric P=O stretching of the phosphodiester groups in nucleic acids, while the broad peak at 1052 cm^–1^ corresponds to the vibrations of the C–O–C and C–O groups in polysaccharide rings.^61,62^

**Figure 5:**
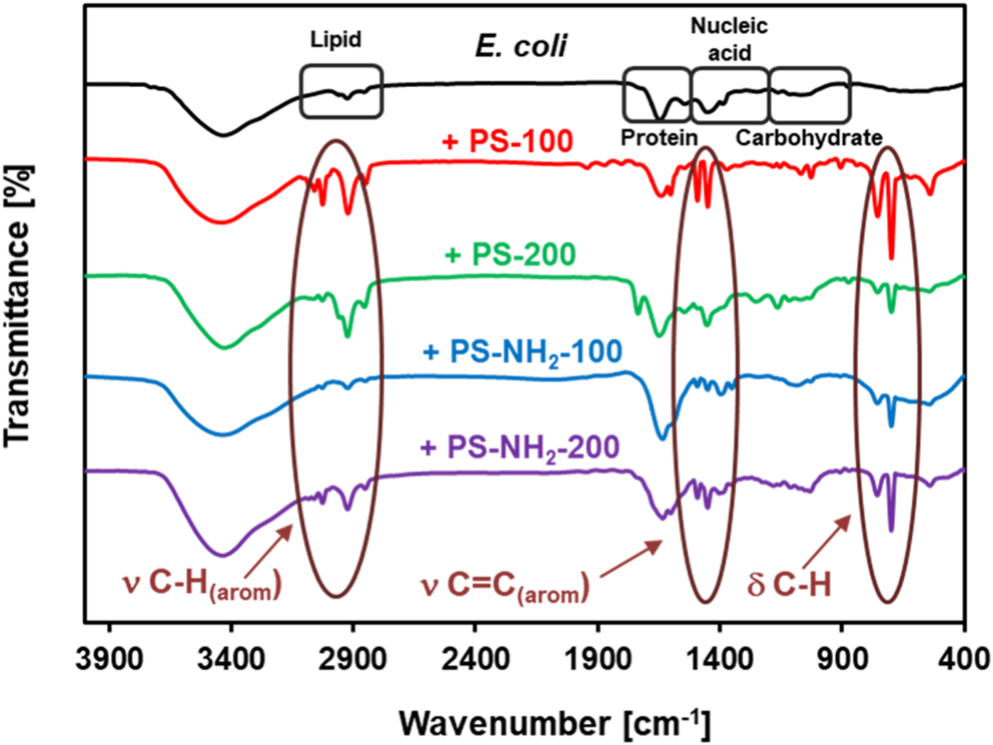
FTIR spectra of *E. coli* strain ATCC 11229 before and after exposure to nonfunctionalized and amino-functionalized polystyrene nanoparticles of different sizes.

Changes in the position and intensity of bands in the FTIR spectrum of *E. coli* caused by its interaction with nanoplastics depend primarily on the size of the polystyrene nanoparticles and their optional surface functionalization. For example, according to Wang et al.,^63^ PS nanoparticles smaller than 50 nm improve the permeability of the *E. coli* cell membrane, while larger particles accumulate mainly on the cell surface.^63–65^ Surface modifications are commonly applied to improve the hydrophilicity and reactivity of PS nanoparticles. The most frequent modifications include amination and carboxylation, which result in a nanoparticle surface with a positive or negative charge. Due to their variable surface charge, PS nanoplastics can interact with proteins on the bacterial cell surface after they have penetrated the cell.^66^

The structure of *E. coli* bacteria after exposure to various sizes of PS and PS-NH_2_ nanoparticles is shown in the FTIR spectra in Figure 5. Characteristic *E. coli* bands are observed in all spectra, confirming the nondestructive effect of PS on bacterial structure, as reported by Zając et al.^40^ The C–O and C=C stretching vibrations, along with the C–H deformation vibrations assigned to the PS nanoparticles,^67^ confirm the presence of plastics in the analyzed samples. The intensity of these bands provides information on the interaction between plastics and bacteria.

FTIR spectra of *E. coli* exposed to polystyrene nanoparticles exhibited changes in the relative intensity of characteristic polystyrene-associated bands compared to control samples. These spectral variations indicate alterations in the local chemical environment at the cell-nanoparticle interface. However, it should be emphasized that reduced band intensity alone does not provide direct evidence of nanoparticle penetration or internalization.

The observed spectral changes may alternatively arise from surface adsorption of nanoparticles, partial masking of infrared-active functional groups, or scattering effects associated with surface-associated particles. Given the ensemble-averaged nature of FTIR measurements, the technique provides indirect information on interfacial interactions but does not resolve the spatial localization of nanoparticles relative to the bacterial envelope.

Spectroscopic observations must therefore be interpreted with caution. In nanoparticle–cell systems, attenuation of characteristic vibrational bands has been widely reported when surface adsorption leads to partial signal shielding rather than true internalization. Consequently, FTIR results in the present study are interpreted as supporting surface-level association between nanoparticles and bacterial cells, without implying penetration across the cell envelope.

Importantly, the FTIR results are qualitatively consistent with the electrokinetic and AFM data, which indicate reversible surface association, partial coverage, and localized adhesion. Together, these findings support a unified interpretation based on physicochemical interactions governed by colloidal forces, rather than on nanoparticle internalization or cytotoxic mechanisms.

Similar cautions against overinterpretation of FTIR data in nanoparticle–cell systems have been emphasized in earlier and more recent studies, highlighting the need for complementary techniques to draw conclusions about nanoparticle localization.^68^

### 3.7 Atomic Force Microscopy (AFM) Evaluation of the Interaction between Bacterial Cells and Nanoparticles

Atomic force microscopy (AFM) was used to evaluate the interaction between bacterial cells and polystyrene nanoparticles in an aqueous environment (Figures 6, S3, and S4). The environmental behavior of polystyrene nanoparticles and their potential interactions with *E. coli* structures were investigated using nonfunctionalized PS nanoparticles (100 and 200 nm) and amino-functionalized PS-NH_2_ nanoparticles at a concentration of 100 μg/mL for 0.5 h.

**Figure 6:**
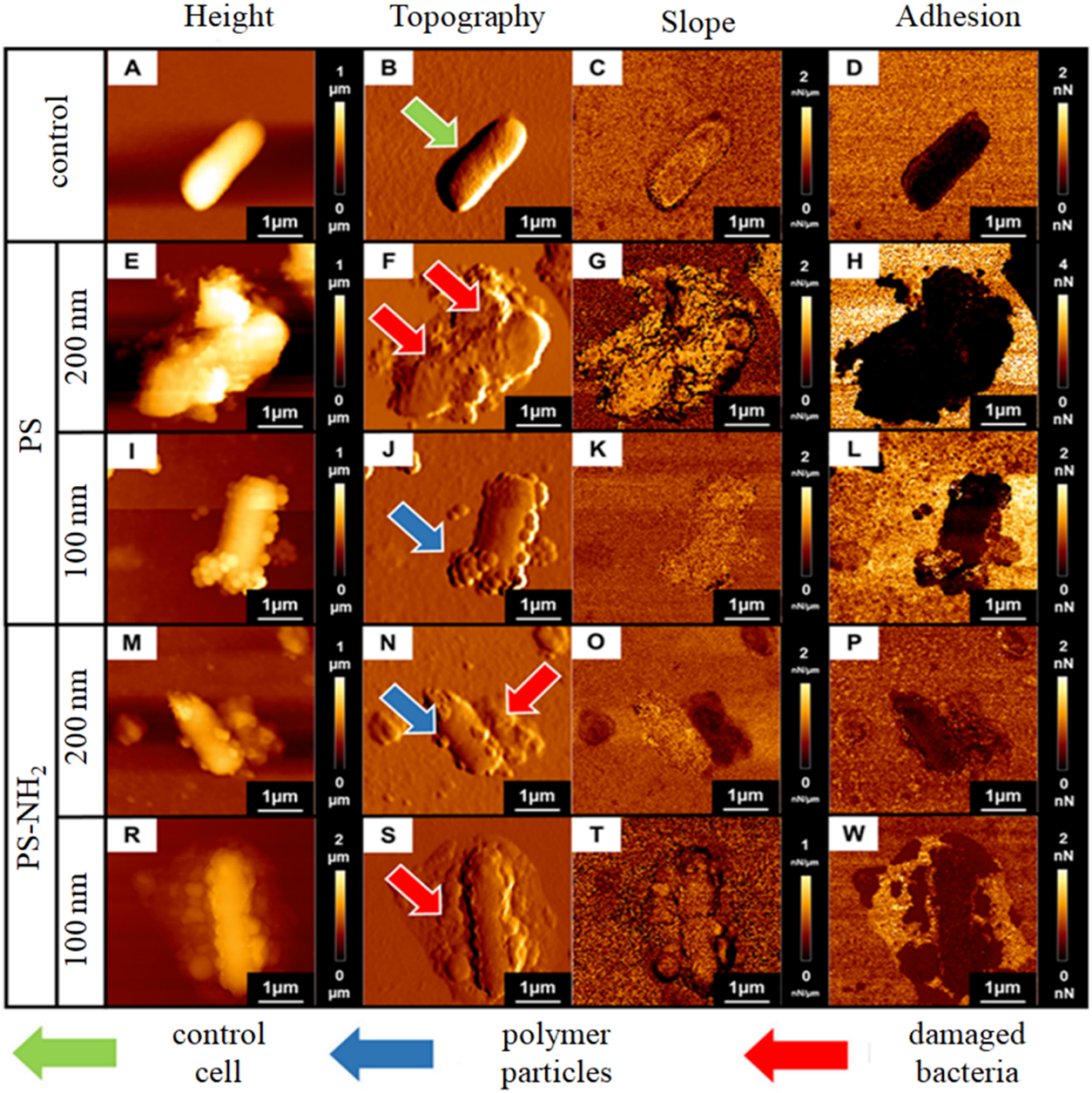
*E. coli* strain ATCC 11229 exposed to 100 and 200 nm nonfunctionalized polystyrene (PS) nanoparticles (panels E–L) and amino-functionalized polystyrene (PS-NH_2_) (panels M–W) at a concentration of 100 μg/mL for 0.5 h, compared to untreated control cells (panels A–D). Panels A, E, I, M, and R display AFM height mode. Panels B, F, J, N, and S show AFM topography in the edge detector function mode. Panels C, G, K, O, and T illustrate the AFM Slope mode, indicating cell stiffness. Panels D, H, L, P, and W depict the AFM Adhesion mode. The green arrow highlights the structure of the control cell. The blue arrows indicate polymeric nanoparticles attached to the bacterial surfaces. Red arrows mark damage to bacterial surfaces. Scale bar - 1 μm.

Figure 6 presents AFM images of *E. coli* cells exposed to PS-100, PS-200, PS-NH_2_-100, and PS-NH_2_-200, compared with untreated control cells. For all tested particles, nanoparticles were observed adhering to the bacterial cell surface, suggesting that polystyrene has an affinity for bacterial structures. The results indicate that even a brief exposure of 0.5 h was sufficient to deposit nanoparticles on the bacterial surface. This effect is particularly evident for 200 nm particles, where particles’ spherical shapes are visible on the bacterial surfaces. Furthermore, AFM imaging revealed localized alterations in surface topography and increased adhesion forces at higher nanoparticle concentrations. These changes were accompanied by morphological alterations of *E. coli*, including surface damage and features suggestive of membrane perturbation. Areas with a high particle density exhibited signs of cellular leakage. The observed changes in *E. coli* cell morphology closely resemble those in *Candida* spp. after exposure to magnetic nanoparticles, as described by Bucki et al.^69^ This finding aligns with our previous work,^40^ where AFM analysis of *S. aureus* strain ATCC 6538 and the *Klebsiella pneumoniae* strain ATCC 4352 exposed to PS NPs revealed no significant morphological alterations.

Where possible, quantitative descriptors such as height variations and adhesion force ranges were extracted. However, in regions with dense nanoparticle coverage, accurate height determination was limited by tip convolution and surface heterogeneity. Adhesion force mapping showed increased local adhesion forces in nanoparticle-exposed samples compared to untreated cells; however, these values should be interpreted as relative indicators, as AFM adhesion measurements depend on probe functionalization, loading force, and hydration state.

At lower nanoparticle concentrations (2 and 20 μg/mL), no detectable changes in bacterial morphology were observed (Figures S3 and S4).

Importantly, the observed morphological features do not provide direct evidence of membrane rupture, cell lysis, or nanoparticle internalization. AFM topography captures only surface-accessible features and cannot resolve subsurface structures. From a physicochemical perspective, the increased surface roughness and adhesion forces are consistent with nanoparticle adsorption onto the bacterial envelope, enhancing van der Waals interactions and partially screening electrostatic repulsion. Such effects are commonly observed in colloidal systems with soft, heterogeneous surfaces and do not necessarily imply irreversible structural damage.

Overall, the AFM observations are consistent with sublethal, surface-level perturbations rather than cytotoxic mechanisms, in agreement with the MIC/MBC results and FTIR analyses.

### 3.8 Physicochemical Interpretation of Bacteria–Nanoparticle Interactions

The interactions between *E. coli* cells and polystyrene nanoparticles observed in this study can be interpreted within a physicochemical framework that accounts for colloidal forces, adsorption phenomena, and the soft, heterogeneous nature of biological interfaces. Although bacterial cells cannot be treated as rigid colloidal particles, classical concepts from colloid and interface science provide a useful qualitative basis for understanding the observed electrokinetic, spectroscopic, and microscopic trends.

Under the low ionic strength conditions employed (0.3 mM NaCl), electrostatic interactions are only weakly screened, resulting in an extended electrical double layer and enhanced sensitivity to surface charge heterogeneities. According to classical DLVO theory, nanoparticle–cell association is governed by the balance between electrostatic repulsion and van der Waals attraction, with the latter becoming increasingly relevant at short separation distances and facilitating reversible surface association.^70–72^ While these simplified conditions enable mechanistic insight into electrostatically driven interactions, the quantitative outcomes should not be directly extrapolated to complex environmental or physiological systems.

Electrokinetic measurements performed in mixed bacteria–nanoparticle suspensions yield an apparent composite signal arising from contributions from bacterial cells, free nanoparticles, and nanoparticles associated with the cell envelope. In such soft and heterogeneous systems, zeta potential values are best interpreted as relative indicators of interfacial electrokinetic changes rather than as direct measures of intrinsic bacterial surface charge. This behavior is consistent with soft-particle electrokinetic theory, in which adsorbed layers shift the effective shear plane and modify electrophoretic mobility without altering the underlying surface charge density.^53,73^

The concentration-dependent shifts in apparent zeta potential, together with their saturation behavior at higher nanoparticle concentrations, are characteristic of an adsorption-limited interaction regime. Increasing nanoparticle concentration enhances the probability of surface association and partial surface coverage, leading to cumulative masking of charged groups and modification of interfacial electrostatic interactions. Similar adsorption-controlled behavior has been reported for colloidal particle interactions with biological and polymeric soft interfaces.^74^

Atomic force microscopy provides complementary nanoscale evidence supporting this physicochemical interpretation. Increased surface roughness and localized adhesion forces observed at higher nanoparticle concentrations are consistent with enhanced van der Waals attraction and partial screening of electrostatic repulsion by surface-associated nanoparticles. Importantly, AFM probes surface-accessible topography and interaction forces and does not resolve subsurface structures; therefore, these observations reflect surface-level association and envelope perturbation rather than membrane rupture or intracellular localization.^75,76^

Spectroscopic changes observed by FTIR can likewise be rationalized within this adsorption-based framework. Attenuation of characteristic vibrational bands may arise from masking of functional groups, changes in local refractive index, or scattering effects associated with nanoparticle coverage, rather than from chemical modification of cellular components or nanoparticle internalization. As emphasized in both classical and recent studies, FTIR spectroscopy of dried samples does not provide direct evidence of intracellular localization and must be interpreted cautiously in nanoparticle–cell systems.^68^

Taken together, the electrokinetic, spectroscopic, and microscopic results converge on a consistent physicochemical picture in which polystyrene nanoparticles interact with *E. coli* cells primarily through reversible, adsorption-driven surface association governed by colloidal forces. This framework explains the observed concentration- and time-dependent trends while remaining entirely consistent with the methodological limitations of the applied techniques and avoiding overinterpretation of biological impact.

## 4 Conclusions

This study provides a physicochemical perspective on the interactions between *E. coli* cells and polystyrene nanoparticles with different surface functionalities under controlled aqueous conditions. By combining electrokinetic, spectroscopic, and microscopic approaches, the work highlights how colloidal forces and interfacial phenomena govern nanoparticle–bacteria interactions at the nanoscale.

Microbiological assays demonstrated concentration-dependent growth inhibition at elevated nanoparticle concentrations, while no definitive evidence of bactericidal or cytotoxic effects was obtained under the applied experimental conditions. These findings underscore the importance of clearly distinguishing between growth inhibition, cell killing, and sublethal effects when evaluating nanoparticle–bacteria systems.

Electrokinetic measurements revealed concentration- and time-dependent shifts in apparent zeta potential; however, these values represent composite electrokinetic signals arising from mixed bacteria–nanoparticle suspensions rather than intrinsic bacterial surface charge. The observed saturation behavior at higher nanoparticle concentrations and rapid equilibration is consistent with an adsorption-limited interaction regime governed by colloidal interactions, including electrostatic screening, van der Waals attraction, and soft-particle behavior, without implying irreversible modification of bacterial surfaces.

Spectroscopic (FTIR) and microscopic (AFM) analyses provided complementary, yet indirect, evidence of nanoparticle surface adhesion and localized envelope perturbations. Importantly, the applied techniques do not offer sufficient spatial resolution to confirm nanoparticle penetration or intracellular internalization, and the data should therefore be interpreted cautiously. Observed changes in FTIR band intensities and AFM-derived surface roughness and adhesion forces are consistent with reversible, surface-level interactions and qualitatively correlate with electrokinetic trends within an adsorption-based physicochemical framework.

Although the experiments were conducted under low ionic strength conditions to enhance electrokinetic sensitivity, the results should be interpreted as mechanistic insights obtained under simplified laboratory conditions rather than as direct representations of nanoparticle behavior in natural or physiological environments.

Overall, this work demonstrates that interactions between polystyrene nanoparticles and *E. coli* cells under the examined conditions are dominated by reversible, surface-level physicochemical processes. The study emphasizes the need for cautious interpretation of electrokinetic, spectroscopic, and microscopic data in complex bio–nano systems. It provides a coherent framework for integrating these techniques without overestimating biological impact.

## Supplementary Material



## Notes

Table S1 (characterization of the polystyrene nanoparticles tested), Figures S1 and S2 (measurements of the zeta potential and particle size of polystyrene nanoparticles as a function of pH), Tables S2 and S3 (zeta potential of *E. coli* cells after exposure to PS and PS-NH_2_ nanoparticles at various concentrations), Tables S4 and S5 (zeta potential of *E. coli* cells after exposure to PS and PS-NH_2_ nanoparticles at different incubation times), and Figures S3 and S4 (AFM images of *E. coli* cells exposed to nonfunctionalized and amino-functionalized polystyrene nanoparticles, respectively, compared to untreated control) (PDF)

## Abbreviations

AFM: atomic force microscopy
ATCC: American Type Culture Collection
DLS: dynamic light scattering
ELS: electrophoretic light scattering
FTIR: Fourier transform infrared spectroscopy
MIC: minimum inhibitory concentration
MBC: minimum bactericidal concentration
NP: nanoplastic
PDI: polydispersity index
PS: nonfunctionalized polystyrene
PS-NH_2_: amino-functionalized polystyrene
ζ: zeta potential (electrokinetic potential)
