# Supplementary material for: Interfacial Interactions between Escherichia coli and Polystyrene Nanoplastics: a Physicochemical Perspective
Source: J Phys Chem B. 2026 Feb 11;130(8):2388–401. doi: 10.1021/acs.jpcb.5c08029 (PMC13298883; doi:10.1021/acs.jpcb.5c08029)
Supplement: Supplementary file 1 [file jp5c08029_si_001.pdf]

Interfacial Interactions between *Escherichia coli* and Polystyrene Nanoplastics: a Physicochemical Perspective

Monika Naumowicz[a,*], Joanna Kotyńska[a], Marcin Zając[b], Piotr Deptuła[c], Joanna Breczko[d], Robert Bucki[c], Izabela Święcicka[e,f]

[a] *Laboratory of Bioelectrochemistry, Department of Physical Chemistry, Faculty of Chemistry, University of Bialystok, 1K K. Ciolkowski Str., 15-245 Bialystok, Poland; joannak@uwb.edu.pl (J.K.), monikan@uwb.edu.pl (M.N.)*

[b] *Doctoral School of Exact and Natural Sciences, University of Bialystok, 1K K. Ciolkowski Str., 15-245 Bialystok, Poland; m.zajac@uwb.edu.pl (M.Z.)*

[c] *Department of Medical Microbiology and Nanobiomedical Engineering, Medical University of Bialystok, 2C A. Mickiewicz Str., 15-222 Bialystok, Poland; piotr.deptula@umb.edu.pl (P.D.), buckirobert@gmail.com (R.B.)*

[d] *Laboratory of Materials Chemistry, Department of Physical Chemistry, Faculty of Chemistry, University of Bialystok, 1K K. Ciolkowski Str., 15-245 Bialystok, 15-245 Bialystok, Poland; j.luszczyn@uwb.edu.pl (J.B.)*

[e] *Department of Microbiology and Biotechnology, Faculty of Biology, University of Bialystok, 1J K. Ciolkowski Str., 15-245 Bialystok, Poland; izabelas@uwb.edu.pl (I.Ś.)*

[f] *Laboratory of Applied Microbiology, Department of Microbiology and Biotechnology, Faculty of Biology, University of Bialystok, 1J K. Ciolkowski Str., 15-245 Bialystok, Poland*

SUPPLEMENTAL FIGURES AND TABLES

Table S1. Characterization of the polystyrene (PS) nanoparticles tested (0.3 mM NaCl, pH = 7.4). PS-NH_2 refers to the amino-modified PS.

Nanoparticle	Size by number [nm]	Size by intensity [nm]	PDI	Zeta potential [mV]
**C_{NPs} = 0.4 µg/ml**				
PS-100	72.51 ± 55.19	263.50 ± 119.20	0.170	-13.70 ± 0.30
PS-200	269.90 ± 82.47	316.90 ± 88.63	0.065	-9.84 ± 0.28
PS-NH_2-100	94.30 ± 36.44	179.00 ± 79.90	0.173	-27.10 ± 1.61
PS-NH_2-200	231.10 ± 67.16	271.20 ± 70.91	0.041	-14.70 ± 0.28
**C_{NPs} = 2 µg/ml**				
PS-100	105.00 ± 34.57	160.60 ± 55.39	0.264	-13.57 ± 1.27
PS-200	211.80 ± 60.22	248.70 ± 65.43	0.089	-14.03 ± 0.90
PS-NH_2-100	172.70 ± 89.40	125.20 ± 36.53	0.508	-23.68 ± 0.83
PS-NH_2-200	209.80 ± 52.08	236.10 ± 52.18	0.010	-15.98 ± 0.65
**C_{NPs} = 20 µg/ml**				
PS-100	100.20 ± 45.23	200.90 ± 85.41	0.146	-13.93 ± 1.50
PS-200	247.60 ± 62.42	273.30 ± 62.33	0.002	-14.55 ± 0.39
PS-NH_2-100	98.17 ± 23.09	123.00 ± 28.02	0.033	-29.93 ± 0.73
PS-NH_2-200	276.90 ± 71.99	309.10 ± 72.87	0.005	-17.98 ± 0.70

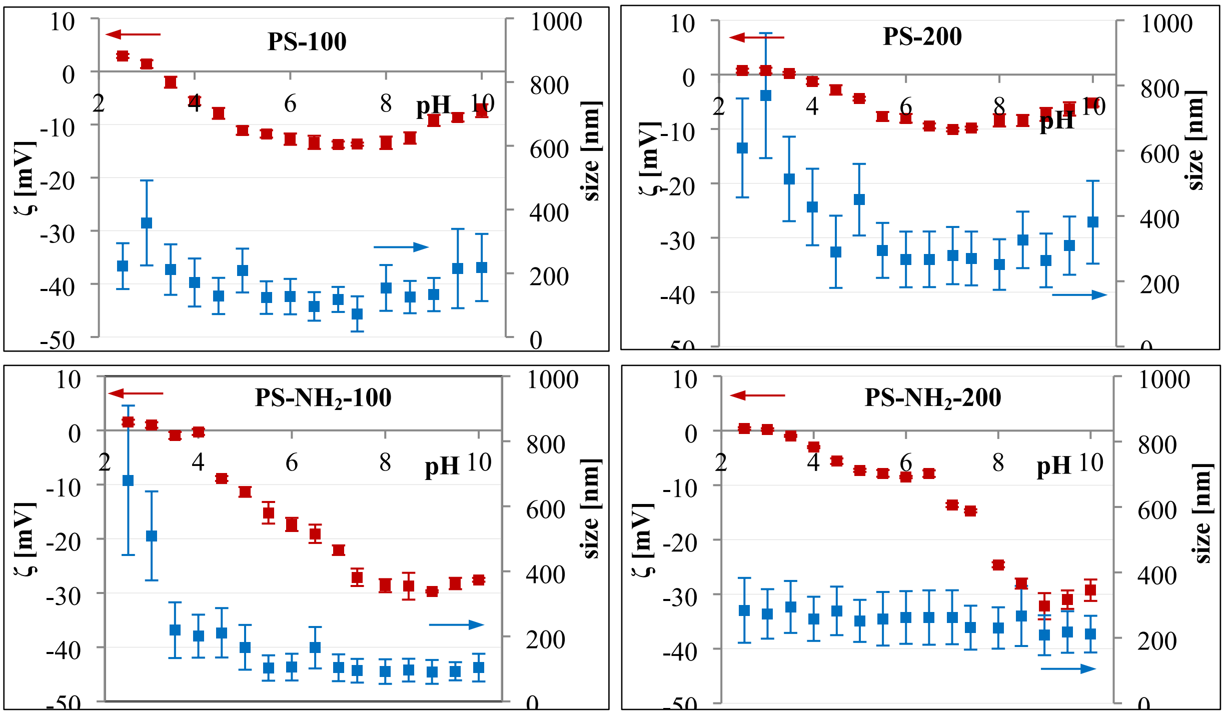

Figure S1. Measurements of zeta potential and particle size of polystyrene nanoparticles (C_{NPs} = 0.4 µg/ml) as a function of pH in 0.3 mM NaCl.

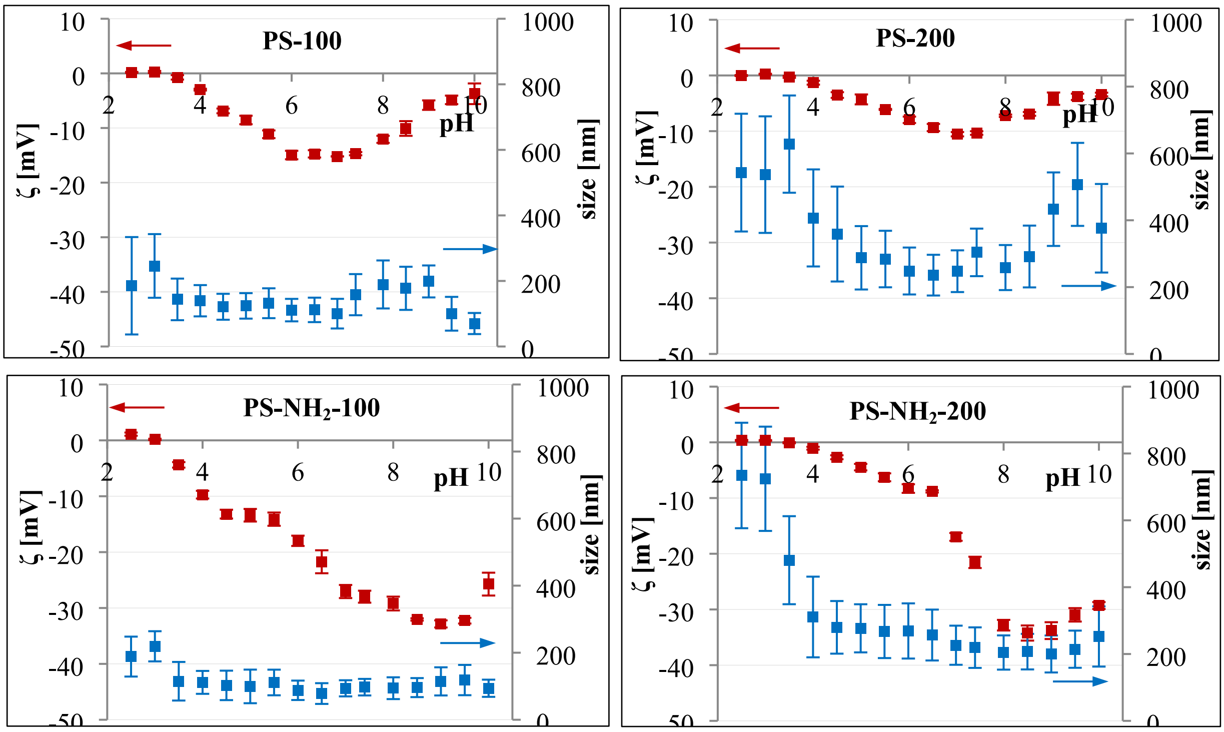

Figure S2. Measurements of zeta potential and particle size of polystyrene nanoparticles (C_{NPs} = 100 µg/ml) as a function of pH in 0.3 mM NaCl.

Table S2. The zeta potential of the *E. coli* cells after exposure to PS nanoparticles (C_{NPs} = 0.4, 2, 20 and 100 μg/ml).

**ζ vs. C**					
**PS-100**					
**pH**	**control**	**0.4**	**2**	**20**	**100**
3	4.18 ± 0.11	1.87 ± 0.31[a]	0.72 ± 0.39[a,b]	0.21 ± 0.69[a,b]	-3.20 ± 1.01[a,b,c,d]
4	-6.76 ± 1.39	-5.62 ± 0.66	-5.57 ± 0.29	-4.90 ± 0.50	-7.49 ± 1.46[a,c,d]
5	-16.49 ± 1.59	-15.03 ± 1.81[a]	-13.37 ± 0.40[a]	-13.03 ± 0.66[a]	-16.38 ± 0.76[a,c,d]
6	-27.08 ± 0.43	-27.45 ± 2.62[a]	-27.90 ± 2.07	-28.10 ± 2.25	-30.25 ± 0.82[a]
7	-34.63 ± 1.26	-29.68 ± 1.57[a]	-29.27 ± 0.58[a]	-33.88 ± 1.47[a,b,c]	-31.43 ± 1.78[a]
8	-35.53 ± 0.94	-30.70 ± 2.46[a]	-33.47 ± 2.81	-34.23 ± -1.96	-31.70 ± 2.38
9	-37.23 ± 1.23	-33.30 ± 0.85	-34.85 ± 1.20	-36.75 ± 1.77	-35.08 ± 1.71
10	-38.85 ± 1.37	-33.95 ± 0.07[a]	-36.00 ± 1.69[b]	-37.45 ± 2.78[a]	-35.98 ± 0.97
11	-39.48 ± 0.66	-37.63 ± 1.23	-39.48 ± 0.66	-36.60 ± 1.81	-36.23 ± 1.49[c]
**PS-200**					
**pH**	**control**	**0.4**	**2**	**20**	**100**
3	4.18 ± 0.11	2.52 ± 0.67[a]	1.51 ± 0.25[a]	0.45 ± 0.36[a,b,c]	-1.85 ± 0.46[a,b,c,d]
4	-6.76 ± 1.39	-3.84 ± 0.53[a]	-1.93 ± 0.82[a,b]	-0.71 ± 0.95[a,b]	-6.58 ± 1.01[b,c,d]
5	-16.49 ± 1.59	-13.15 ± 0.78[a]	-12.10 ± 0.28[a,b]	-12.00 ± 0.91[a]	-20.52 ± 0.99[a,b,c,d]
6	-27.08 ± 0.43	-27.55 ± 1.63	-24.60 ± 1.56[a,b]	-22.97 ± 1.16[a,b]	-31.50 ± 0.71[a,b,c,d]
7	-34.63 ± 1.26	-27.73 ± 1.47[a]	-27.53 ± 2.08[a]	-27.18 ± 0.84[a]	-31.97 ± 1.23[a,b,c,d]
8	-35.53 ± 0.94	-29.90 ± 3.10[a]	-27.55 ± 1.48[a]	-28.08 ± 1.09[a]	-35.77 ± 2.11[b,c,d]
9	-37.23 ± 1.23	-32.70 ± 2.52[a]	-27.90 ± 2.40[a]	-30.73 ± 1.86[a]	-36.40 ± 2.11[c,d]
10	-38.85 ± 1.37	-34.00 ± 2.43[a]	-28.55 ± 1.77[a,b]	-32.68 ± 2.40[a]	-37.60 ± 1.70[c]
11	-39.48 ± 0.66	-32.55 ± 1.99[a]	-30.77 ± 1.82[a]	-37.68 ± 1.69[b,c]	-38.33 ± 2.41[b,c]

[a] Statistically significant differences vs. control group, $p < 0.05$;

[b] Statistically significant differences vs. modified *E. coli* exposed to PS NP_s (C = 0.4 μg/ml), $p < 0.05$; [c] Statistically significant differences vs. modified *E. coli* exposed to PS NP_s (C = 2 μg/ml), $p < 0.05$;

[d] Statistically significant differences vs. modified *E. coli* exposed to PS NP_s (C = 20 μg/ml), $p < 0.05$.

Table S3. The zeta potential of the *E. coli* cells after exposure to $PS\text{-}NH_2$ nanoparticles (C_{NPs} = 0.4, 2, 20 and 100 μg/ml).

**ζ vs. C**					
**$PS\text{-}NH_2$-100**					
**pH**	**control**	**0.4**	**2**	**20**	**100**
3	4.18 ± 0.11	-0.86 ± 1.32[a]	-0.08 ± 0.91[a]	-2.44 ± 1.59[a]	-2.22 ± 0.29a
4	-6.76 ± 1.39	-5.81 ± 0.80	-4.28 ± 0.19	-3.92 ± 0.84	-10.18 ± 0.35[a,b,c,d]
5	-16.49 ± 1.59	-13.27 ± 3.58	-10.05 ± 0.64[a]	-10.85 ± 0.92[a]	-24.83 ± 0.90[a,b,c,d]
6	-27.08 ± 0.43	-24.53 ± 2.81	-23.15 ± 1.20[a]	-22.50 ± 1.95[a]	-34.18 ± 0.70[a,b,c,d]
7	-34.63 ± 1.26	-28.98 ± 1.33[a]	-28.98 ± 1.77[a]	-24.25 ± 1.66[a,b,c]	-35.58 ± 0.94[b,c,d]
8	-35.53 ± 0.94	-30.23 ± 1.86[a]	-30.95 ± 0.49[a]	-28.73 ± 2.27[a]	-36.80 ± 1.23[b,c,d]
9	-37.23 ± 1.23	-31.40 ± 1.48[a]	-31.60 ± 0.70[a]	-29.00 ± 1.36[a]	-39.20 ± 0.95[b,c,d]
10	-38.85 ± 1.37	-31.97 ± 3.15[a]	-31.30 ± 1.39[a]	-28.50 ± 2.21[a]	-39.48 ± 1.82[b,c,d]
11	-39.48 ± 0.66	-33.23 ± 1.88[a]	-31.87 ± 0.64[a]	-28.80 ± 2.26[a]	-30.00 ± 2.27[b,c,d]
**$PS\text{-}NH_2$-200**					
**pH**	**control**	**0.4**	**2**	**20**	**100**
3	4.18 ± 0.11	0.23 ± 0.44[a]	0.11 ± 0.38[a]	-0.07 ± 0.63[a]	-0.66 ± 0.09[a]
4	-6.76 ± 1.39	-3.97 ± 1.80	-3.72 ± 0.62[a]	-6.01 ± 0.24	-8.65 ± 0.61[c]
5	-16.49 ± 1.59	-15.00 ± 1.27	-14.52 ± 0.49	-13.05 ± 0.33	-32.25 ± 0.37[a,b,c,d]
6	-27.08 ± 0.43	-25.06 ± 1.64	-24.52 ± 1.75[a]	-20.42 ± 1.26[a,b,c]	-35.61 ± 1.33[a,b,c,d]
7	-34.63 ± 1.26	-33.65 ± 2.51[a]	-30.25 ± 1.71[a,b]	-29.41 ± 0.44[a,b,c]	-38.35 ± 2.10[a,b,c,d]
8	-35.53 ± 0.94	-35.52 ± 0.49	-33.93 ± 1.44	-33.07 ± 1.57	-39.98 ± 1.50[a,b,c,d]
9	-37.23 ± 1.23	-37.06 ± 1.86	-34.68 ± 1.50[a,b]	-32.25 ± 0.79[a,b,c]	-40.13 ± 0.94[a,b,c,d]
10	-38.85 ± 1.37	-37.51 ± 1.30	-35.38 ± 1.40[a,b]	-35.59 ± 1.39[a,b]	-40.73 ± 1.53[a,b,c,d]
11	-39.48 ± 0.66	-39.49 ± 1,03	-36.60 ± 2.19	-34.67 ± 1.99[a,b]	-39.88 ± 2.04

[a] Statistically significant differences vs. control group, $p < 0.05$;
[b] Statistically significant differences vs. modified *E. coli* exposed to $PS\text{-}NH_2$ NP_s (C = 0.4 μg/ml), $p < 0.05$;
[c] Statistically significant differences vs. modified *E. coli* exposed to $PS\text{-}NH_2$ NP_s (C = 2 μg/ml), $p < 0.05$;
[d] Statistically significant differences vs. modified *E. coli* exposed to $PS\text{-}NH_2$ NP_s (C = 20 μg/ml), $p < 0.05$.

Table S4. The zeta potential of the *E. coli* cells after exposure to PS nanoparticles (t = 0.5, 1 and 3 h).

**ζ vs. t**				
**PS-100**				
**pH**	**control**	**0.5**	**1**	**3**
3	4.18 ± 0.11	-2.87 ± 1.00[a]	-2.29 ± 1.36[a]	-1.41 ± 0.65[a]
4	-6.76 ± 1.39	-7.19 ± 1.14	-6.17 ± 1.00	-5.73 ± 0.60
5	-16.49 ± 1.59	-12.10 ± 1.43[a]	-13.57 ± 2.47	-12.43 ± 0.72
6	-27.08 ± 0.43	-17.15 ± 1.50[a]	-25.05 ± 2.33	-27.20 ± 0.44
7	-34.63 ± 1.26	-27.60 ± 0.87[a]	-28.17 ± 1.15[a]	-27.93 ± 0.75[a]
8	-35.53 ± 0.94	-30.08 ± 0.88[a]	-30.33 ± 1.19[a]	-31.35 ± 1.63[a]
9	-37.23 ± 1.23	-32.68 ± 1.50[a]	-34.35 ± 1.19[a]	-31.87 ± 0.67[a]
10	-38.85 ± 1.37	-33.73 ± 1.19[a]	-35.58 ± 1.25[a,b]	-31.80 ± 1.20[a,b,c]
11	-39.48 ± 0.66	-34.53 ± 1.05[a]	-36.53 ± 0.67[a]	-33.57 ± 0.91[a]
**PS-200**				
**pH**	**control**	**0.5**	**1**	**3**
2	4.18 ± 0.11	-1.27 ± 1.75[a]	-1.71 ± 0.60[a]	-1.74 ± 0.37[a]
3	-6.76 ± 1.39	-4.71 ± 0.87	-5.13 ± 0.79	-6.16 ± 1.30
4	-16.49 ± 1.59	-6.64 ± 1.30[a]	-9.15 ± 0.45[a]	-6.48 ± 1.21[a,b]
6	-27.08 ± 0.43	-10.41 ± 2.29[a]	-23.27 ± 2.15[a,b]	-18.33 ± 0.35[a,b]
7	-34.63 ± 1.26	-16.40 ± 1.49[a]	-26.60 ± 2.97[a,b]	-23.65 ± 1.91[a,b]
8	-35.53 ± 0.94	-22.35 ± 2.24[a]	-28.93 ± 1.07[a,b]	-26.55 ± 0.07[a,b,c]
9	-37.23 ± 1.23	-31.25 ± 1.48[a]	-30.23 ± 1.75[a]	-28.57 ± 1.01[a]
10	-38.85 ± 1.37	-33.35 ± 1.77[a]	-31.80 ± 1.55[a]	-31.60 ± 2.51[a]
11	-39.48 ± 0.66	-30.55 ± 2.57[a]	-33.15 ± 1.94[a]	-34.20 ± 1.43[a]

[a] Statistically significant differences vs. control group, $p < 0.05$;
[b] Statistically significant differences vs. modified *E. coli* exposed to PS NP_s (t = 0.5 h) $p < 0.05$;
[c] Statistically significant differences vs. modified *E. coli* exposed to PS NP_s (t = 1 h), $p < 0.05$.

Table S5. The zeta potential of the *E. coli* cells after exposure to PS-NH_2 nanoparticles (t = 0.5, 1 and 3 h).

ζ **vs. t**				
**PS-NH2-100**				
**pH**	**control**	**0.5**	**1**	**3**
3	4.18 ± 0.11	-1.53 ± 0.89[a]	-1.79 ± 0.37[a]	-0.67 ± 0.32[a]
4	-6.76 ± 1.39	-7.80 ± 1.93	-7.09 ± 0.33	-8.23 ± 1.13
5	-16.49 ± 1.59	-24.30 ± 1.50[a]	-26.95 ± 0.19[a]	-24.08 ± 0.17[a,c]
6	-27.08 ± 0.43	-28.93 ± 1.58	-34.70 ± 1.58[a,b]	-32.50 ± 2.21[a,b,c]
7	-34.63 ± 1.26	-33.25 ± 2.40	-34.63 ± 1.43	-35.10 ± 2.21
8	-35.53 ± 0.94	-35.38 ± 1.46	-36.60 ± 1.36	-36.53 ± 1.96
9	-37.23 ± 1.23	-38.73 ± 1.71	-36.78 ± 1.17	-38.50 ± 1.81
10	-38.85 ± 1.37	-38.98 ± 2.05	-39.60 ± 1.62	-40.73 ± 0.81
11	-39.48 ± 0.66	-40.53 ± 1.43	-39.78 ± 1.16	-42.43 ± 2.01
**PS-NH_2-200**				
**pH**	**control**	**0.5**	**1**	**3**
2	4.18 ± 0.11	-4.72 ± 0.60[a]	-0.35 ± 0.52[a,b]	-0.59 ± 0.08[a,b]
3	-6.76 ± 1.39	-27.75 ± 0.67[a]	-6.98 ± 0.09[b]	-6.18 ± 0.36[b]
4	-16.49 ± 1.59	-34.65 ± 1.40[a]	-29.00 ± 0.62[a,b]	-29.35 ± 0.90[a,b]
6	-27.08 ± 0.43	-34.50 ± 0.96[a]	-34.15 ± 1.53[a]	-33.63 ± 1.58[a]
7	-34.63 ± 1.26	-35.78 ± 1.29	-33.40 ± 1.84	-35.85 ± 1.40
8	-35.53 ± 0.94	-36.20 ± 2.34	-37.45 ± 1.02	-37.00 ± 1.32
9	-37.23 ± 1.23	-36.47 ± 1.88	-37.95 ± 0.74	-38.88 ± 0.54
10	-38.85 ± 1.37	-38.18 ± 0.84	-38.48 ± 1.71	-38.78 ± 1.62
11	-39.48 ± 0.66	-39.00 ± 1.50	-39.30 ± 1.73	-40.13 ± 1.65

[a] Statistically significant differences vs. control group, $p < 0.05$;
[b] Statistically significant differences vs. modified *E. coli* exposed to PS-NH_2 NP_s (t = 0.5 h), $p < 0.05$;
[c] Statistically significant differences vs. modified *E. coli* exposed to PS-NH_2 NP_s (t = 1 h), $p < 0.05$.

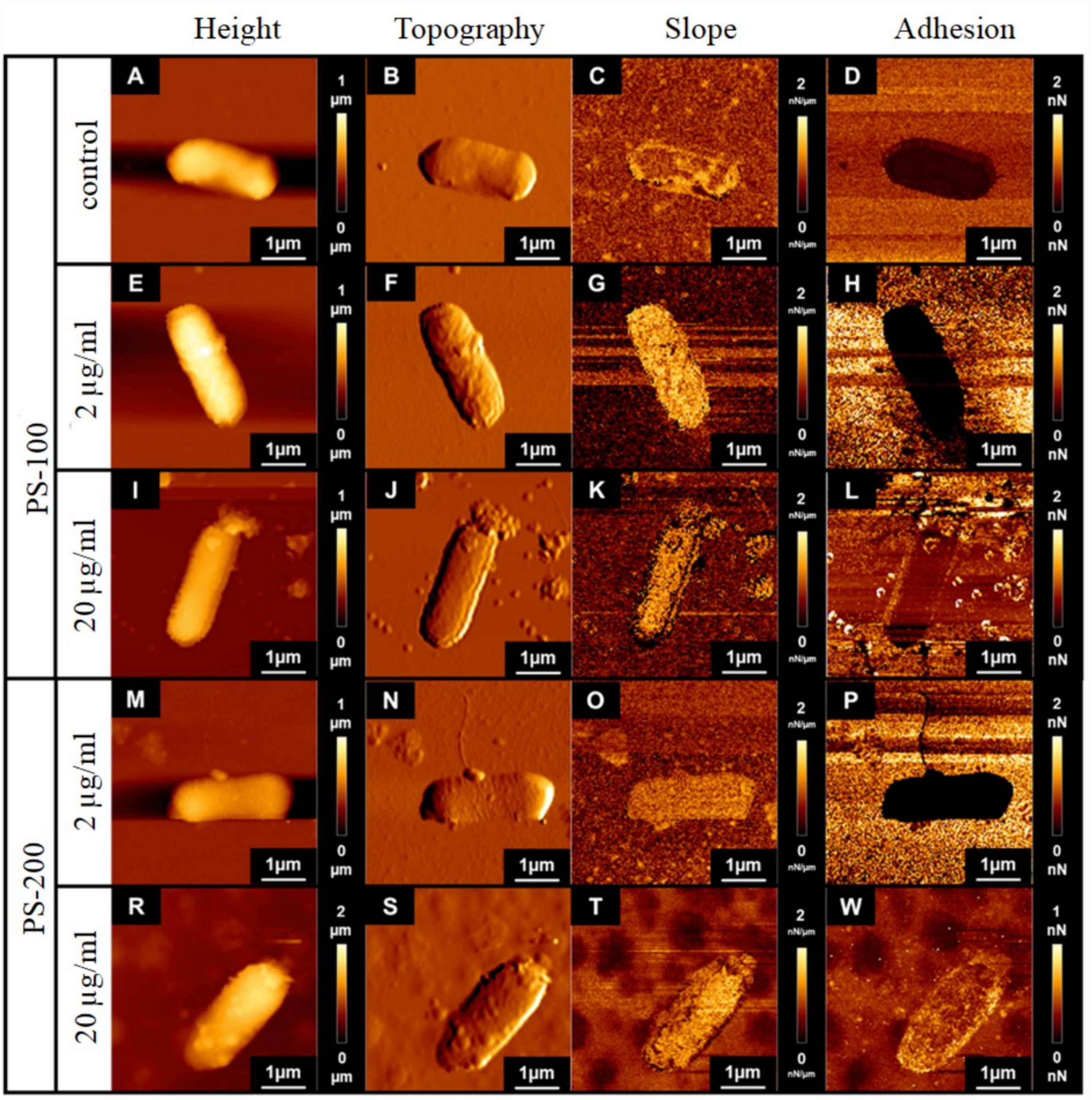

Figure S3. *E. coli* strain (ATCC 11229) exposed to 100 nm and 200 nm non-functionalized polystyrene (PS) nanoparticles at concentrations of 2 µg/mL (panels E - H and M - P) and 20 µg/mL (panels I – L and R - W) for 0.5 h, compared to untreated control (A - D). Panels A, E, I, M, and R display AFM Height mode. Panels B, F, J, N, and S illustrate AFM Topography mode with an edge detector function. Panels C, G, K, O, and T depict AFM Slope mode, indicating cell stiffness. Panels D, H, L, P, and W show AFM Adhesion mode. Scale bar- 1µm.

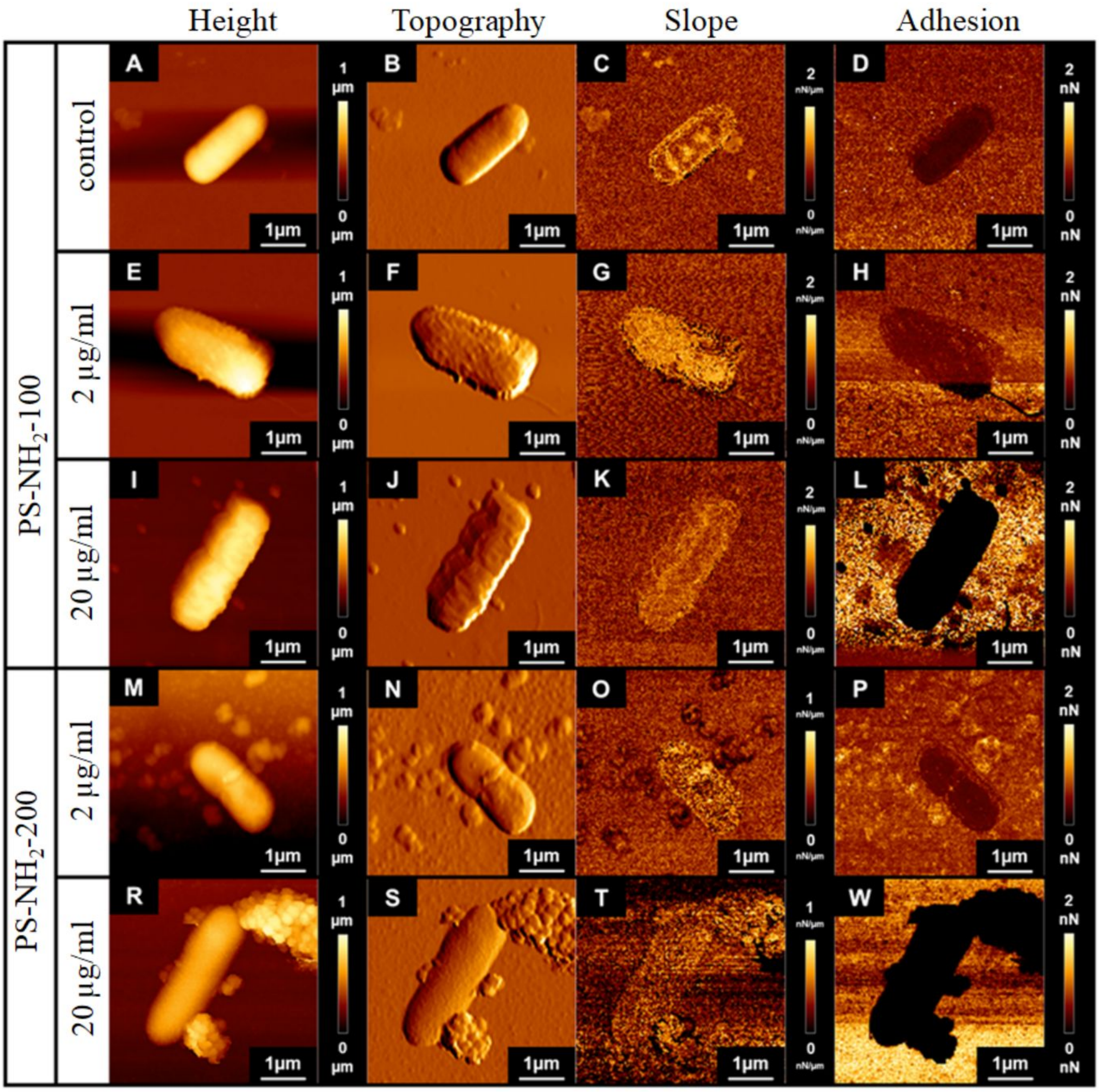

Figure S4. *E. coli* strain (ATCC 11229) exposed to 100 nm and 200 nm amino-functionalized ($PS\text{-}NH_2$) nanoparticles at concentrations of 2 µg/mL (panels E - H and M - P) and 20 µg/mL (panels I – L and R - W) for 0.5 h, compared to untreated control (A - D). Panels A, E, I, M, and R display AFM Height mode. Panels B, F, J, N, and S illustrate AFM Topography mode with an edge detector function. Panels C, G, K, O, and T depict AFM Slope mode, indicating cell stiffness. Panels D, H, L, P, and W show AFM Adhesion mode. Scale bar- 1µm.
